# How Sucrose Preference Is Gained and Lost: An In-Depth Analysis of Drinking Behavior during the Sucrose Preference Test in Mice

**DOI:** 10.1523/ENEURO.0195-23.2023

**Published:** 2023-09-26

**Authors:** Andreas B. Wulff, Phylicia Cooper, Emmanuela Kodjo, Eliana Abel, Scott M. Thompson

**Affiliations:** 1 Department of Physiology, University of Maryland School of Medicine, Baltimore, MD 21201; 2 Program in Neuroscience, Graduate Program in Life Sciences, University of Maryland Baltimore, Baltimore, MD 21201; 3 ASCEND Scholars Program, Morgan State University, Baltimore, MD 21251; 4 Department of Psychiatry, University of Colorado School of Medicine, Aurora, CO 80045

**Keywords:** stress, reward, depression, lickometry, anhedonia

## Abstract

The sucrose preference test (SPT) is a widely used preclinical assay for studying stress-sensitive reward behaviors and antidepressant treatments in rodents, with some face, construct, and predictive validity. However, while stress-induced loss of sucrose preference is presumed to reflect an anhedonic-like state, little detail is known about what behavioral components may influence performance in the SPT in stress-naive or stressed rodents. We analyzed the licking microstructure of mice during the SPT to evaluate how preference is expressed and lost following chronic stress. In stress-naive mice, preference is expressed as both longer and more numerous drinking bouts at the sucrose bottle, compared with the water bottle. We also found evidence that memory of the sucrose bottle location supports preference. Through manipulations of the caloric content of the sweetener or caloric need of the mouse, we found that energy demands and satiety signals do not affect either preference or the underlying drinking behavior. Both acute and chronic stress impaired sucrose location memory and reduced the number of drinking bouts at the sucrose bottle, the latter of which explained the loss of sucrose preference in stress susceptible mice compared with stress resilient mice. Female mice generally exhibited similar drinking behavior to male mice but may be less susceptible to chronic stress and display better memory performance than male mice, both before and after chronic stress. Our data suggest that chronic stress inhibits a sucrose preference by reducing reward seeking behavior without affecting palatability.

## Significance Statement

The sucrose preference test (SPT) is a widely used behavioral assay of reward and hedonic state. Here, we provide a detailed behavioral analysis of drinking behavior during the test in male and female mice and reveal that the overall test results depend on multiple behavioral components, including rapid feedback related to palatability, reward seeking, and memory of reward location. These behaviors were largely independent of caloric content or sex of the mouse. Chronic stress lowered sucrose preference by reducing reward seeking, but not palatability.

## Introduction

Depression is a devastating mental illness affecting 5% of the world’s population yearly (Global Burden of Disease Collaborative Network, 2021). Understanding brain mechanisms underlying symptoms of depression is crucial for improving treatments. Rodent models of depressive symptoms are important in these efforts as they allow us to study and target the neural, synaptic, and genetic changes associated with depressive-like behaviors.

Many rodent behaviors have been used to study depression-relevant pathology and antidepressant efficacy. Some of these, including the commonly used forced swim and tail suspension tests or learned helplessness test, have been criticized for lack of face validity and, in recent years, their use have been discouraged in favor of behaviors with better validity, such as behaviors that probe responses to rewarding stimuli, like the sucrose preference test (SPT; Bale et al., 2019; Reardon, 2019; Scannell et al., 2022).

In the SPT, rodents choose between drinking regular tap water or a low concentration sucrose solution. Stress-naive rodents have a strong preference for the sucrose solution but this preference is diminished or lost following chronic stress. This stress-induced behavioral change resembles anhedonia, a core symptom of human depression. Importantly, preference for sucrose is restored by chronic (approximately two weeks) but not acute (24–48 h) administration of selective serotonin reuptake inhibitors (SSRIs; LeGates et al., 2018), thus mimicking the delayed response of human depressive symptoms to SSRIs.

Results in the SPT are typically reported as the percentage of the total liquid consumed that was from the sucrose solution (Liu et al., 2018). It is widely assumed that loss of sucrose preference after chronic stress results from the sucrose solution being less rewarding, i.e., it reflects a change in hedonic state, particularly when accompanied by changes in other reward behaviors. Overall drinking behavior has many components, however, not all associated with hedonic processing. Sucrose preference may be expressed through immediate responses to the sweet taste and motivation to seek out the sweet solution. Motivation may be affected by satiety signals or caloric need. Finally, expression of sucrose preference may be supported by memory of the presence and location of the sucrose solution. Lickometry has been used to study the drinking behavior of mice when presented with sweet and caloric solutions (for review, see Johnson, 2018), but these experiments are usually performed in stress-naive rodents and in contexts that do not involve a choice. While some studies have used lickometry during the SPT, only total licking has been reported (Tye et al., 2013; Cerniauskas et al., 2019). Further, these experiments have often been performed in water-deprived or food-deprived rodents, which could itself affect drinking behaviors. Upon completion of the project reported here, Verharen and colleagues (2023) published a paper performing lickometry during a sucrose preference test with untrained mice in operant chambers under food-deprived conditions. They used computational modeling to distinguish learning and hedonic components of sucrose consumption and found that chronic stress reduced hedonic components of sucrose consumption whereas acute stress affected the learning rate.

In parallel to the experiments of Verharen et al. (2023), we also used lickometry to dissect the components of drinking behavior that underlie the expression of sucrose preference in stress naive and chronically stressed mice in their home cages under nonfood deprived conditions. We found that mice expressed sucrose preference through several distinct drinking behaviors related to palatability, reward seeking, and reward memory. These behaviors were largely independent of caloric content or sex of the mouse. Finally, we found that impaired reward seeking following chronic stress seemed to largely mediate the loss of sucrose preference in stress-susceptible mice. Our findings are generally consistent with those of Verharen and colleagues; however, there are noteworthy differences in methodology and results that we will expand on in Discussion.

## Materials and Methods

### Animals

All procedures were approved by the University of Maryland Baltimore Animal Use and Care Committee and were conducted in full accordance with the National Institutes of Health *Guide for the Care and Use of Laboratory Animals*.

Male and female C57Bl/6J mice were acquired from The Jackson Laboratory. Upon delivery, mice were transferred to the room where experiments were performed and housed for a week before beginning experiments. Mice were eight weeks of age on initiating experiments. Mice were housed on 12/12 h light/dark cycle (lights on at 7 A.M., lights off at 7 P.M.) in a standard Plexiglas mouse cage with *ad libitum* access to autoclaved chow (LabDiet, 5010) and water. The vivarium was kept at 20–23°C. Upon initiation of experiments mice were singly housed for the duration of the experiments.

### Lickometry setup and analysis

We used an inexpensive lickometry system described in Hayar et al. (2006) to record the drinking behavior of mice during the SPT. In brief, an aluminum plate was placed toward the front of the home-cage with a wire connecting the metal plate to the ground of an AD converter (Molecular Devices Digidata, Molecular Devices). Two bottles were placed at the front end of the home-cage in custom-designed and 3D-printed bottle holders. Bottles were made from 50-ml falcon tubes that were cut and had a plastic valve that was secured in the screw cap of the tube (Extended Data Fig. 1-1). After filling bottles with water or sweet solutions, Parafilm was used to close the top and a wire was inserted into the bottle connecting the solution to the signal input of an AD converter. When the mouse stands on the aluminum plate and drinks from the bottle, the circuit is completed, and a junction potential created between the mouse and the liquid is recorded as a positive deflection with an amplitude of ∼400 mV. The setup was designed for wires to be connected between the aluminum plate/bottles and the AD converter at all times while allowing the lid to tightly close on the homecage. Thus, the setup could be kept in the homecage throughout the experiments and the same device was reused for each mouse.

A total of 380 mouse x night lickometry recordings were performed. Out of these, 49 recordings (12.9%) were excluded because of bottle leaking (20 recordings), setup-to-digitizer connection issues (21 recordings), and software malfunction (8 recordings). Connection issues were caused by the aluminum plate being covered by bedding, uninsulated wires causing crossed or dissipated signals, excessive tension on wires causing dissipation of signals, or lost connection between wire and aluminum plate. The frequency of these specific issues was not recorded.

**Figure 1.**
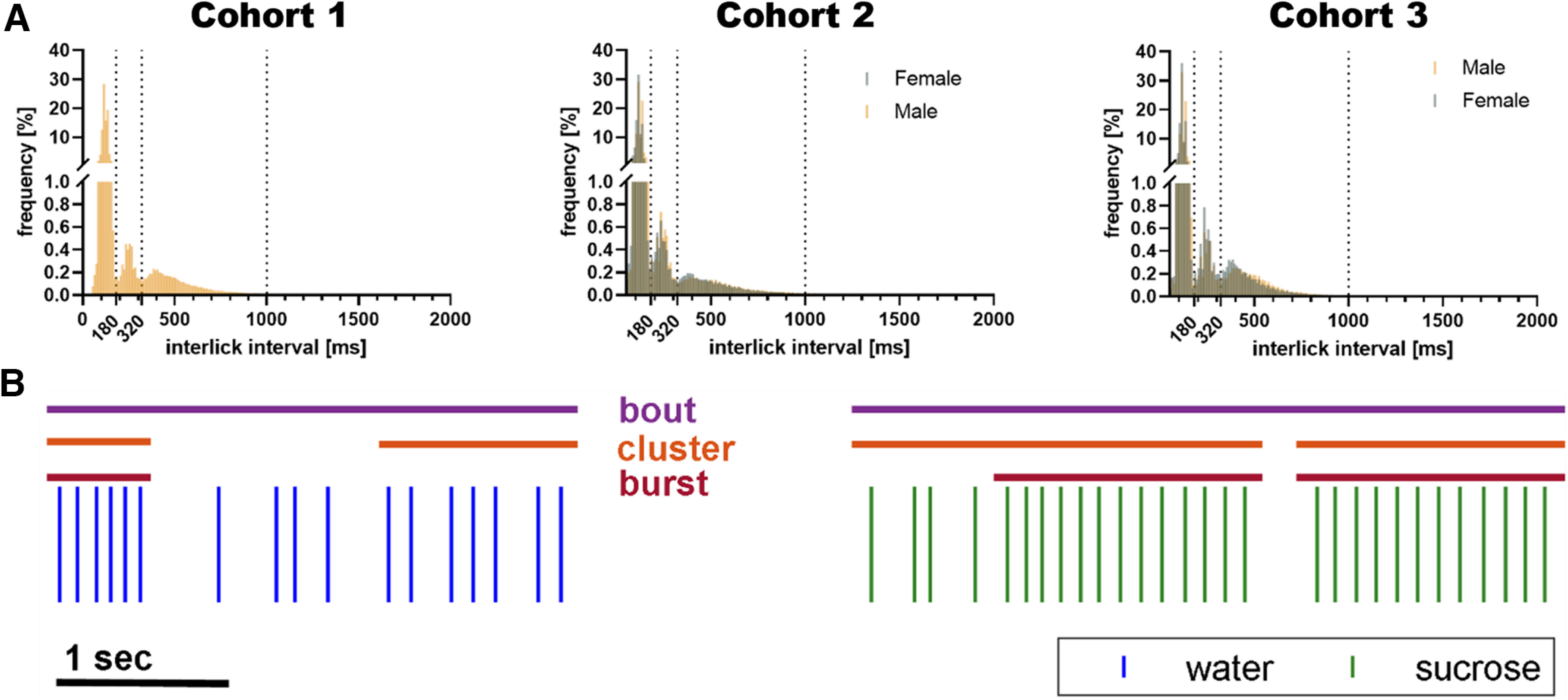
Licks can be grouped into bursts, clusters, and bouts based on interlick interval. ***A***, Histograms of the interlick interval (ILI) reveal three distinct groups of licks that are comparable among all three cohorts and across sexes. The large majority of licks have ILIs < 180 ms as delimited by the first dotted line. The second group have ILIs between 180 and 320 ms located between the first and second dotted line. The last group of licks have ILIs between 320 and ∼1000 ms. These three different groups can be defined as bursts, clusters, and bouts, respectively. ***B***, A representative example of a raster plot from a mouse during the sucrose preference is shown below the graphs. Each vertical line represents a lick at either the water bottle (blue) or sucrose bottle (green). Horizontal lines above the raster plot indicates whether the licks based on their ILI can be considered to belong to a burst (red), cluster (orange), or bout (purple).

Previous studies on licking microstructure have identified three readily distinguishable groups based on interlick intervals (ILIs): bursts, licks with ILIs of <250 ms; clusters, licks with ILIs between 250 and 500 ms; and pauses, licks with ILIs over 500 ms, during which rodents engage in other behaviors (Davis and Smith, 1992). We created histograms of the ILIs from our three experiments and as expected observed three distinguishable groups of licks, with ILIs (1) <180 ms, (2) 180–320 ms, (3) >320 ms (Fig. 1*A*). In mice, these ILIs are faster than those in rats and are consistent with previous reports in C57Bl/6 mice (90% of licks occur with an ILI of <160 ms; Boughter et al., 2007; St. John et al., 2017). We further observed that the large majority of licks (>90%) belong to the first group which is consistent with previous observations (Davis and Smith, 1992). The three categories are likely explained as the “miss” of one lick (clusters) or two licks (pauses) from a normal drinking burst. In order to quantify drinking behavior independently from lickometer fidelity or fine-motor lick function, we grouped licks with ILIs <1000 ms as lick bouts, defined as four or more licks with ILIs <1000 ms (Fig. 1*B*), as done previously and having been shown to reflect subjective preference (Yang et al., 2020) and in some instances showing superior sensitivity to differences in drinking behavior than either bursts or clusters (Johnson et al., 2010).

10.1523/ENEURO.0195-23.2023.f1-1 Extended Data Figure 1-1 Lickometry setup. Image depicts the in-cage component of the lickometry setup used to record drinking behavior of mice during the sucrose preference test. Bottles were made from 50-ml falcon tubes with the ends cut off and plastic sippers inserted into the screw lid. A custom-designed 3D-printed bottle holder was used to secured the bottles to the side of the mouse cages in a way that allowed the cages to be completely closed during the testing. An aluminum plate was placed at the bottom of the mouse cages under the bottles with a wire connected to the ground of the digitizer. A wire was connected from the inside of each bottle to the signal inputs of a digitizer. Additionally, cages contained standard bedding not depicted in the image. Download Figure 1-1, TIFF file.

Signals were digitized using pClamp software and exported for offline analysis using custom scripts in MATLAB. A primary analysis was performed to detect individual licks. Recordings were loaded into MATLAB using ABFLoad (Collman, 2020; https://github.com/fcollman/abfload), and licks were detected using peak detection (prominence >80 mV, width 15–80 ms). Licks with interlick intervals (ILIs) of <50 ms were labeled as double-peaks resulting from noise and excluded. To determine whether lickometers had accurately captured the drinking behavior of mice overnight, correlations were made between the liquid consumed from each bottle and the total amount of licking at the bottle overnight. The data from an individual mouse from that night was excluded if a bottle had leaked overnight, as determined by visual inspection, or if it was manually determined that the lickometer had performed insufficiently (usually seen as detecting too few licks resulting from disrupted connection between the cage and the AD converter or because of crossover of signals between inputs resulting from poor isolation). Lickometer performance was determined via visual identification of outliers in a correlation plot and subsequently confirmed through inspection of the raw lickometry signal. These analyses took place blinded to the contents of the bottle or the condition of the mouse. The resulting correlation between liquid consumed and the total amount of licking reveal a strong correlation (*R*^2^ > 0.8; Extended Data Fig. 1-2*A*) for all experiments, indicating that the lickometer accurately captured the drinking behavior for subsequent analyses. When estimating this correlation between experimental conditions within experiments, no difference was observed in the slope of the correlation (Extended Data Fig. 1-2*B*).

**Figure 2.**
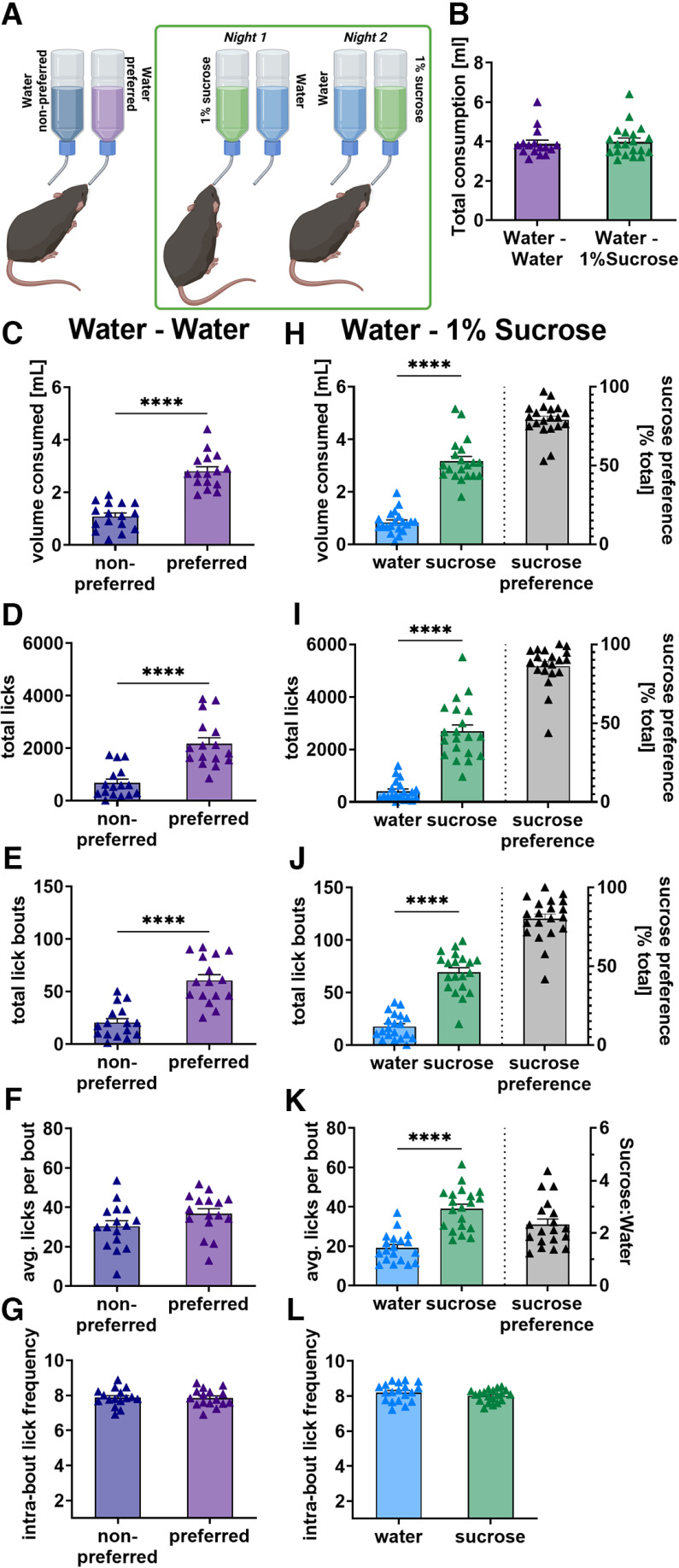
Sucrose preference is expressed via increased number of licks, lick bouts and licks per bout. ***A***, Mice were habituated with two bottles containing water before testing mice with one bottle containing sucrose over two nights. The location of the bottle was switched between the two nights and drinking behavior was averaged over the two nights. ***B***, Addition of a sucrose option did not affect the total consumption across the two bottles (*W* = 25, *p* = 0.5). ***C–E***, When sucrose is absent mice still drink more from one preferred bottle (purple) than another (blue) which can be observed in the volume consumed (***C***, *t* = 7.22, *p* < 0.0001), total licks (***D***, *W* = 132, *p* < 0.0001) and number of lick bouts (***E***, *t* = 5.86, *p* < 0.0001). ***F***, ***G***, No difference is seen between preferred and unpreferred water bottle in licks per bout (***F***, *t* = 1.92, *p* = 0.07) or intrabout lick frequency (***G***, *t* = 0.29, *p* = 0.78). ***H–L***, When sucrose is present, mice drink more from the sucrose bottle (green) than water bottle (blue) resulting in a robust expression of sucrose preference (gray). This is seen in volume consumed (***H***, *W* = 210, *p* < 0.0001), total licks (***I***, *W* = 208, *p* < 0.0001), number of lick bouts (***J***, *t* = 10.33, *p* < 0.0001), and licks per bout (***K***, *t* = 9.05, *p* < 0.0001) but not intrabout lick frequency (***L***, *t* = 1.82, *p* = 0.09). *p < 0.05, **p < 0.01, ***p < 0.001, ****p < 0.0001.

10.1523/ENEURO.0195-23.2023.f1-2 Extended Data Figure 1-2 Capacitive lickometer accurately captures the drinking behavior of mice during the sucrose preference across sex and behavioral setting. Correlations between total licks and volume consumed were performed for all included recording sessions. ***A***, In all cohorts, this correlation was highly significant (*p* < 0.0001) with an *R*^2^ = 0.83 in cohort 1, *R*^2^ = 0.85 in cohort 2, and *R*^2^ = 0.90 in cohort 3. ***B***, The slope of this correlation was not significantly different between different experimental sessions either for (***D***) cohort 1 (*F*_(2,228)_ = 1.55, *p* = 0.21), (***E***) cohort 2 (*F*_(7,578)_ = 1.890, *p* = 0.07), or (***F***) cohort 3 (*F*_(3,656)_ = 2.041, *p* = 0.11). Download Figure 1-2, TIFF file.

In secondary analyses using a custom MATLAB script, we identified and counted the number of lick bouts, the average number of licks per bout, and the average frequency of lick bouts in 30 min intervals for the duration of the recording. Another custom script was used to count the number of times each bottle was approached after a pause of >5 min (return to bottle) and the number of times mice switched from one bottle to the other with an ILI of <60 s (quick switch).

### Sucrose preference test

Mice were habituated to two bottles containing tap water in their homecage with free access for a full night. Their contents were weighed individually at the beginning and end to determine how much liquid had been consumed from each bottle. We used these results to determine the side preference, based on the bottle from which the mice drank the most. For the second night, mice were trained to recognize sucrose solution. A solution consisting of tap water containing sucrose (Domino Sugar) at a concentration of 2% w/v replaced the tap water in the least preferred bottle and mice were allowed to freely drink from either bottle for one night with the volume consumed from each bottle determined as previously described. Following the training night, the position of the bottles was switched, and the sucrose solution was exchanged for a 1% sucrose solution. The amount consumed from each bottle was then measured over two nights with the position of the bottles switched between the two nights. A sucrose preference was calculated for each night. The final sucrose preference was calculated as the average of the sucrose preference over the two test nights. For subsequent SPTs with the same mice, tests with 1% sucrose solution were performed immediately without prior re-training.

In some experiments, 1% sucrose was substituted for a noncaloric sweetener (PURE, 0.1% w/v). SPT was performed while mice had *ad libitum* access to food. However, in some experiments, food was mildly restricted (∼50% of normal consumption) during the SPT night followed by normal *ad libitum* access to food at the conclusion of each SPT night. Mice were allowed at least 3 d of normal housing conditions between each altered SPT testing.

### Multimodal stress

We used a multimodal restraint stress protocol, which has been shown to reduce sucrose preference in mice and increase the stress hormone corticosterone (Hesselgrave et al., 2021; Cole et al., 2022; Troppoli et al., 2022). Mice were restrained in tubes (Midwest Scientific, Fenton, MO or custom-designed, 3D printed) and simultaneously subjected to loud white noise, strobe lights, ∼30° cage tilting, and predator odor (fox urine, Trap Shack Company, Neillsville, WI). This was continued for 4 h/d (starting at 9 A.M. and ending at 1 P.M.) for either 1 d (acute stress) or daily for two to three weeks (chronic stress). SPT was started 4 h after the end of acute stress or 29 h after ending the last session of chronic stress. Following chronic stress, analysis was performed separately on mice resilient to stress (>70% sucrose preference poststress) and mice susceptible to stress (<70% sucrose preference poststress). The 70% cutoff point for discriminating between susceptible and resilient mice was chosen as this cutoff is 2–3 SDs below the mean sucrose preference observed in stress-naive mice based on historical behavioral data from our lab (data not shown).

### Experimental design and statistical analyses

The reported data arises from three separate experiments.

**Figure 3.**
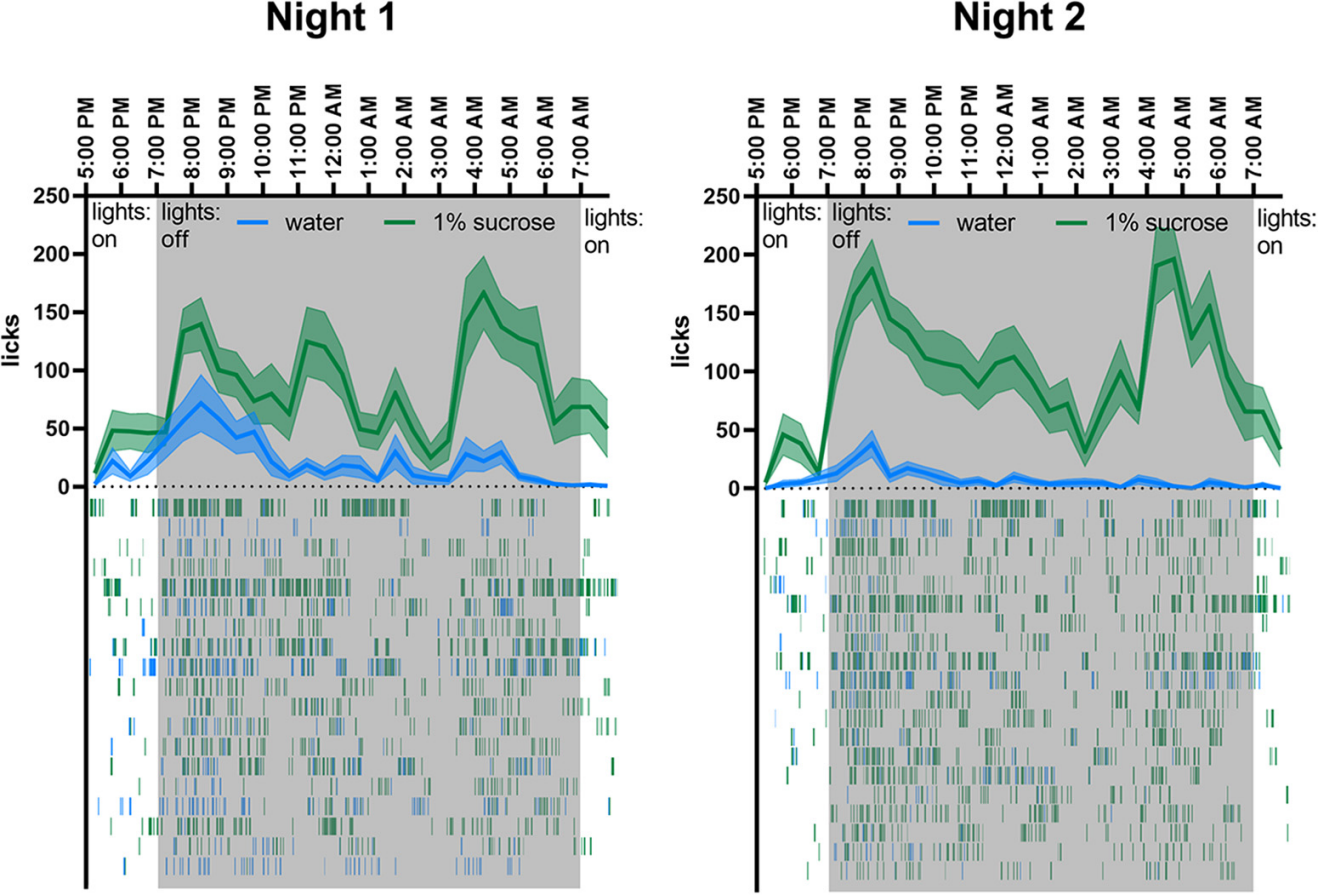
Temporal analysis of drinking reveals circadian-like pattern of drinking during the SPT. Line graph shows average drinking of water (blue) and 1% sucrose (green) during the two sessions of SPT (light color band indicates SE). The raster plots below show the individual licks (vertical lines) for each individual animal in rows. Upon entering the dark phase (gray) at 7:00 P.M., a spike in drinking is observed which slowly decline resulting in a sustained pause from drinking from 1–3 A.M. before a second spike in drinking is observed at 4:00 A.M. *N* = 19 mice for night 1, *N* = 20 mice for night 2.

**Figure 4.**
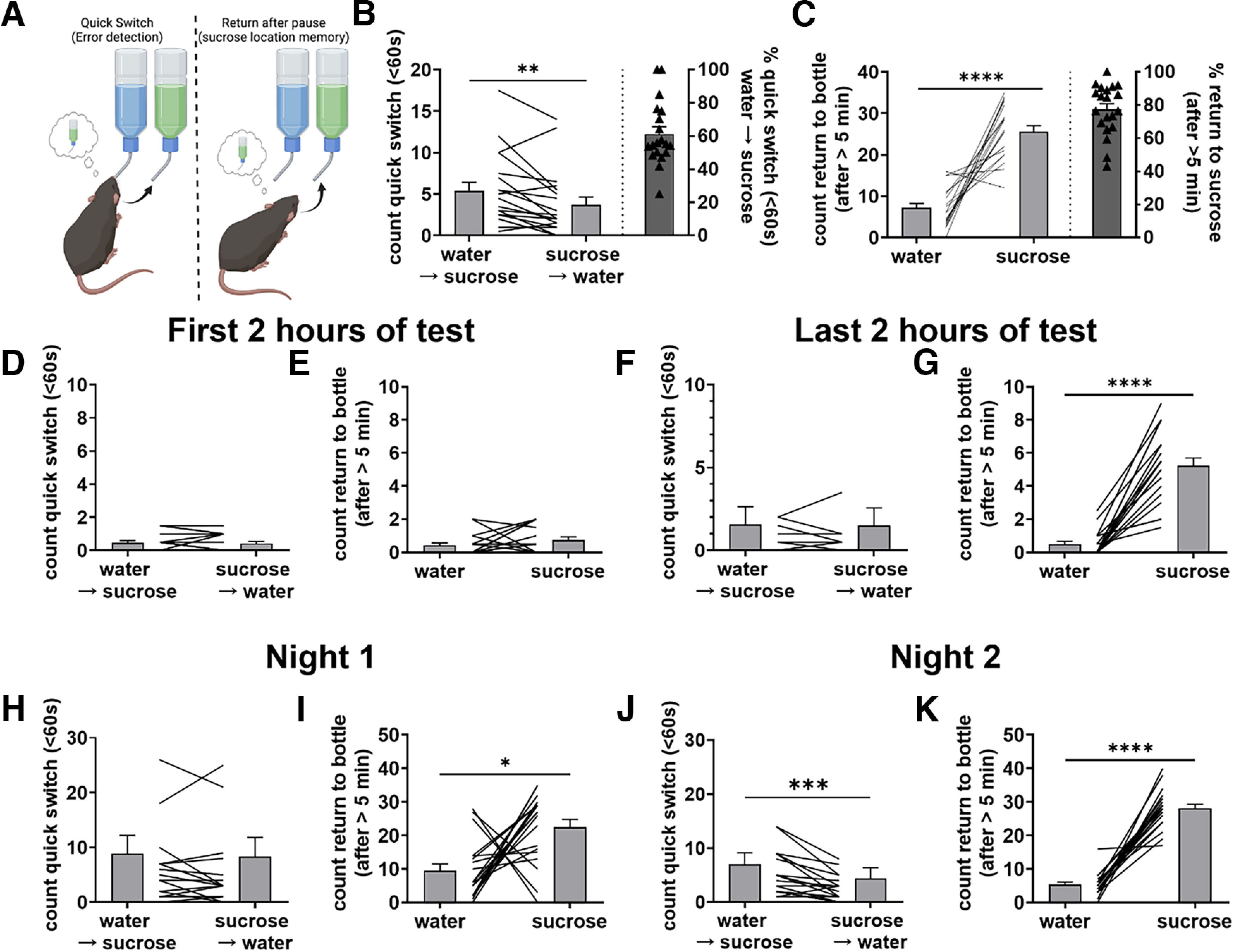
Evidence of error correcting and sucrose location memory in drinking behavior. ***A***, To examine the influence of memory during the SPT, we measured the number of quick switches (<60 s) from water to sucrose as an estimate of error detection, and the number of returns to the sucrose bottle following a >5-min pause from drinking as an estimate of sucrose location memory. ***B***, ***C***, Averaged over the two nights of testing, mice performed more quick switches from water to sucrose than from sucrose to water (***B***, *W* = −111, *p* = 0.006) and returned more to the sucrose bottle than the water bottle following a >5-min pause from drinking (***C***, *t* = 7.90, *p* < 0.0001). ***D–G***, Averaged over the two nights, during the first 2 h of testing, mice did not differ in number of quick switches between bottles (***D***, *W* = −7, *p* = 0.77) or in number of returns to either bottle following a pause from drinking (***E***, *W* = 35, *p* = 0.195) and during the last 2 h of testing mice did not differ in number of quick switches between bottles (***F***, *W* = −2, *p* = 0.94) but did return more to the sucrose bottle than water bottle following a pause from drinking (***G***, *W* = 210, *p* < 0.0001). ***H–K***, On night 1, mice did not show difference in number of quick switches between bottles (***H***, *W* = −30, *p* = 0.38) but did return more to the sucrose bottle than water bottle (***I***, *W* = 124, *p* = 0.011). On night 2, mice performed more quick switches from water to sucrose than from sucrose to water (***J***, *W* = −124, *p* = 0.0004) and returned more often to the sucrose bottle than the water bottle following a pause from drinking (***K***, *W* = 210, *p* < 0.0001). *p < 0.05, **p < 0.01, ***p < 0.001, ****p < 0.0001.

**Figure 5.**
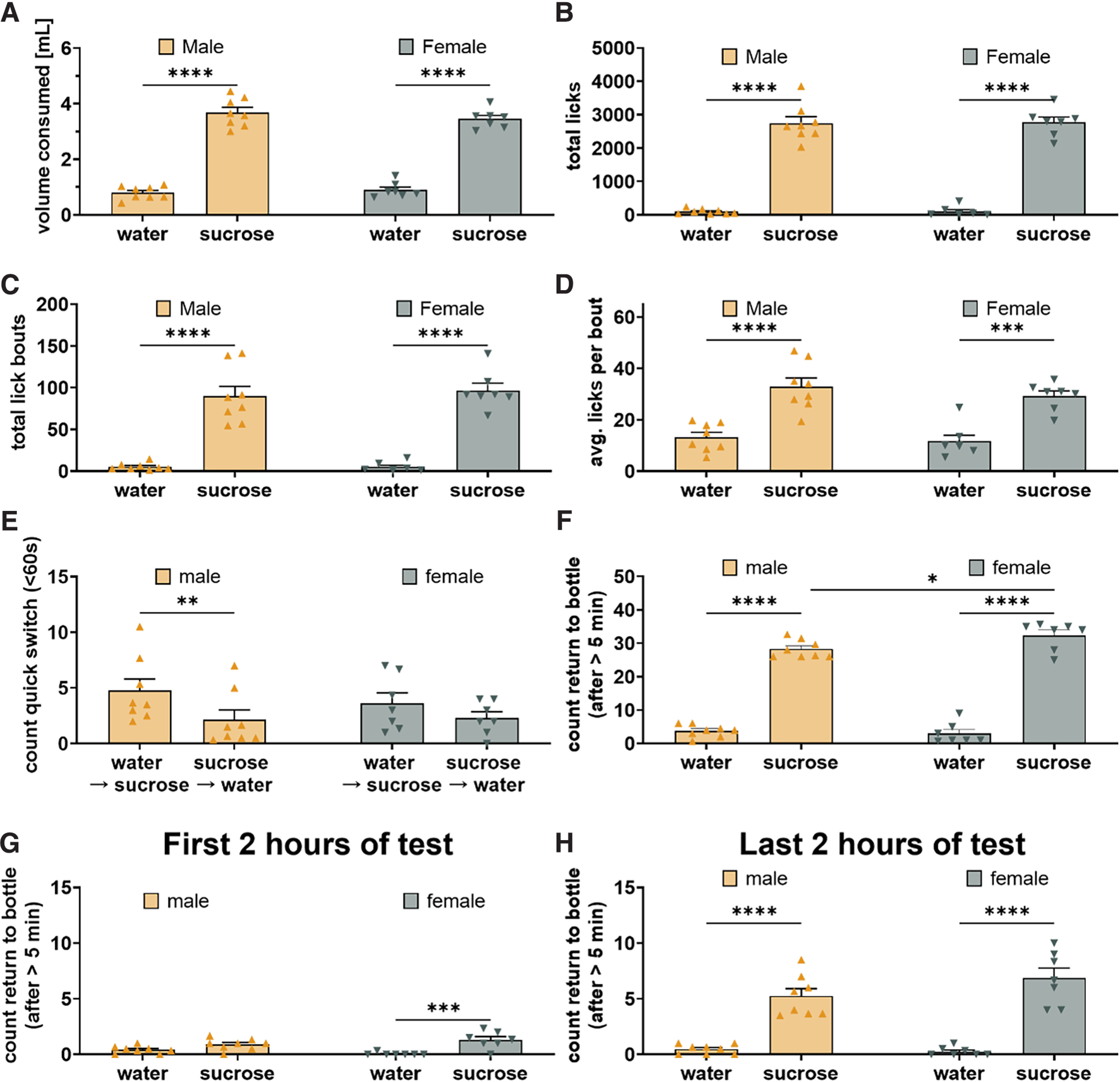
Male and female mice exhibit comparable drinking-behavior during SPT with 1% sucrose. Both sexes drink more 1% sucrose than water as seen in volume consumed (***A***, *F*_(1,13)_ = 421.3, *p* < 0.0001; males *t* = 15.95, *p* < 0.0001; females *t* = 13.18, *p* < 0.0001), total licks (***B***, *F*_(1,13)_ = 379.8, *p* < 0.0001; males *t* = 14.20, *p* < 0.0001; females *t* = 13.40, *p* < 0.0001), lick bouts (***C***, *F*_(1,13)_ = 120.5, *p* < 0.0001; males *t* = 7.70, *p* < 0.0001; females *t* = 7.83, *p* < 0.0001), and licks per bout (***D***, *F*_(1,13)_ = 68.71, *p* < 0.0001; males *t* = 6.42, *p* < 0.0001; females *t* = 5.35, *p* = 0.0003). Mice performed more quick switches (<60 s) from water to sucrose than from sucrose to water largely driven by performance in male mice (***E***, *F*_(1,13)_ = 21.23, *p* = 0.0005; males *t* = 4.47, *p* = 0.0013; females *t* = 2.125, *p* = 0.104). Sex significantly affected the ratio with which mice returned to the sucrose bottle and water bottle following a >5-min pause from drinking (***F***, *F*_(1,13)_ = 4.76, *p* = 0.048) with both male and female mice returning significantly more to the sucrose bottle than the water bottle (males *t* = 15.88, *p* < 0.0001; females *t* = 17.84, *p* < 0.0001) but females returning more to the sucrose bottle than males (*t* = 2.64, *p* = 0.028). During the first 2 h of the night we observed a trending effect of sex on the ratio with which mice returned to the sucrose and water bottle (***G***, *F*_(1,13)_ = 4.341, *p* = 0.0575) with female mice returning significantly more to the sucrose bottle than water bottle which is not observed for male mice (males *t* = 1.87, *p* = 0.16; females *t* = 4.60, *p* = 0.0010). During the last 2 h of testing mice return more to the sucrose bottle than water bottle following a pause from drinking (***H***, *F*_(1,13)_ = 125.8, *p* < 0.0001; males *t* = 6.89, *p* < 0.0001; females *t* = 8.91, *p* < 0.0001). *p < 0.05, **p < 0.01, ***p < 0.001, ****p < 0.0001.

**Figure 6.**
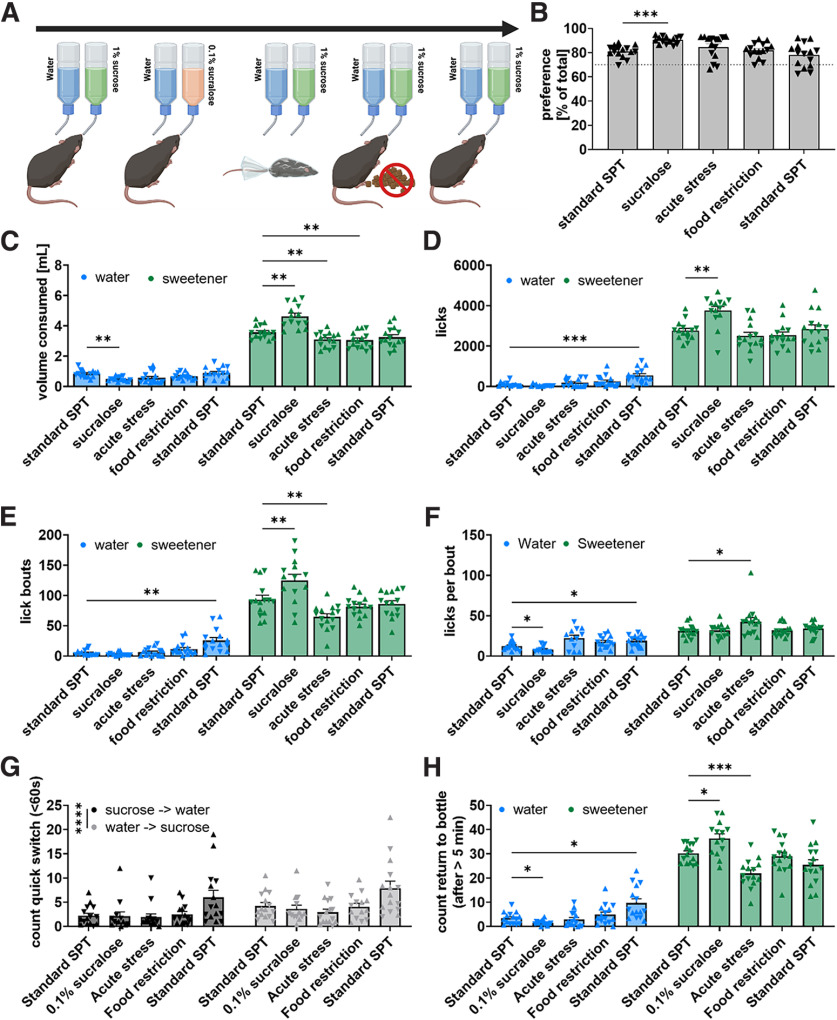
Little effect of caloric content but wide-ranging effects of acute stress on drinking behavior in male and female mice. ***A***, Following a standard SPT mice underwent three preference tests to measure effects of caloric content and acute stress on drinking behavior. First, 1% sucrose was replaced with 0.1% noncaloric sucralose. Second, sucrose preference was tested on nights following acute multimodal stress. Third, sucrose preference was tested while food was restricted to ∼50% of normal consumption. Finally, mice were re-tested on the standard SPT. ***B***, Mice exhibited an increased preference for 0.1% sucralose than 1% sucrose over water (***B***, Friedman test *p* < 0.0001, *Z* = 3.94, *p* = 0.0003) but neither acutes stress nor food-restriction affected sucrose preference. ***C***, ***D***, Experimental manipulations also affected the ratio of sucrose versus water drinking as seen in volume consumed (***C***, *F*_(3.197,43.15)_ = 33.63, *p* < 0.0001) and total licks (***D***, *F*_(2.774,37.45)_ = 12.53, *p* < 0.0001) with mice consuming more sweetener and less water when sucrose is replaced with sucralose (***C***, water *t* = 4.52, *p* = 0.0023; sweetener *t* = 4.91, *p* = 0.0011), consumed less sucrose when subjected to acute stress or mild food restriction (***C***, stress *t* = 3.71, *p* = 0.0093; food *t* = 3.69, *p* = 0.0097), exhibited more licks at the water bottle on retesting under standard conditions (***D***, *t* = 4.92, *p* = 0.0009), and more licks at the sweet bottle when sucrose was replaced with sucralose (***D***, *t* = 4.82, *p* = 0.0013). ***E***, Experimental manipulations also affected the number of lick bouts mice initiated at the water or sweet bottle (***E***, *F*_(2.969,40.08)_ = 23.44, *p* < 0.0001) producing an increased number of lick bouts at the water bottle on retesting under standard conditions (***E***, *t* = 4.31, *p* = 0.0029) increasing lick bouts at the sweet bottle when replacing sucrose with sucralose (***E***, *t* = 4.14, *p* = 0.0046) and reducing lick bouts at the sucrose bottle after acute stress (***E***, *t* = 3.95, *p* = 0.0058). ***F***, Across all experimental manipulations, female mice produced fewer licks per bout than male mice at the sweet bottle (data not shown; *F*_(1,13)_ = 6.69, *p* = 0.023) but experimental manipulations affected licks per bout at the water or sweet bottle independently of sex (*F*_(2.14,24.66)_ = 3.699, *p* = 0.037) reducing licks per bout at the water bottle when replacing sucrose with sucralose (***F***, *t* = 3.11, *p* = 0.036), increasing licks per bout at the sucrose bottle following acute stress (***F***, *t* = 2.87, *p* = 0.048) and increasing licks per bout at the water bottle on retesting under standard conditions (***F***, *t* = 3.09, *p* = 0.032). ***G***, Across all experimental manipulations, mice performed more quick switches (<60 s) from water to sucrose than sucrose to water (***G***, *F*_(1,14)_ = 35.99, *p* < 0.0001). ***H***) Experimental manipulations affected memory performance (***H***, *F*_(2.864,38.67)_ = 18.94, *p* < 0.0001) where replacing sucrose with sucralose resulted in reduced returns to water and increased returns to sucralose following a >5-min pause (***H***, water *t* = 3.05, *p* = 0.037; sucralose *t* = 3.06, *p* = 0.036), acute stress reduced returns to the sucrose bottle (***H***, *t* = 5.44, *p* = 0.0003), and on retesting under standard conditions we observed an increased number of returns to the water bottle (***H***, *t* = 3.47, *p* = 0.015). *p < 0.05, **p < 0.01, ***p < 0.001, ****p < 0.0001.

**Figure 7.**
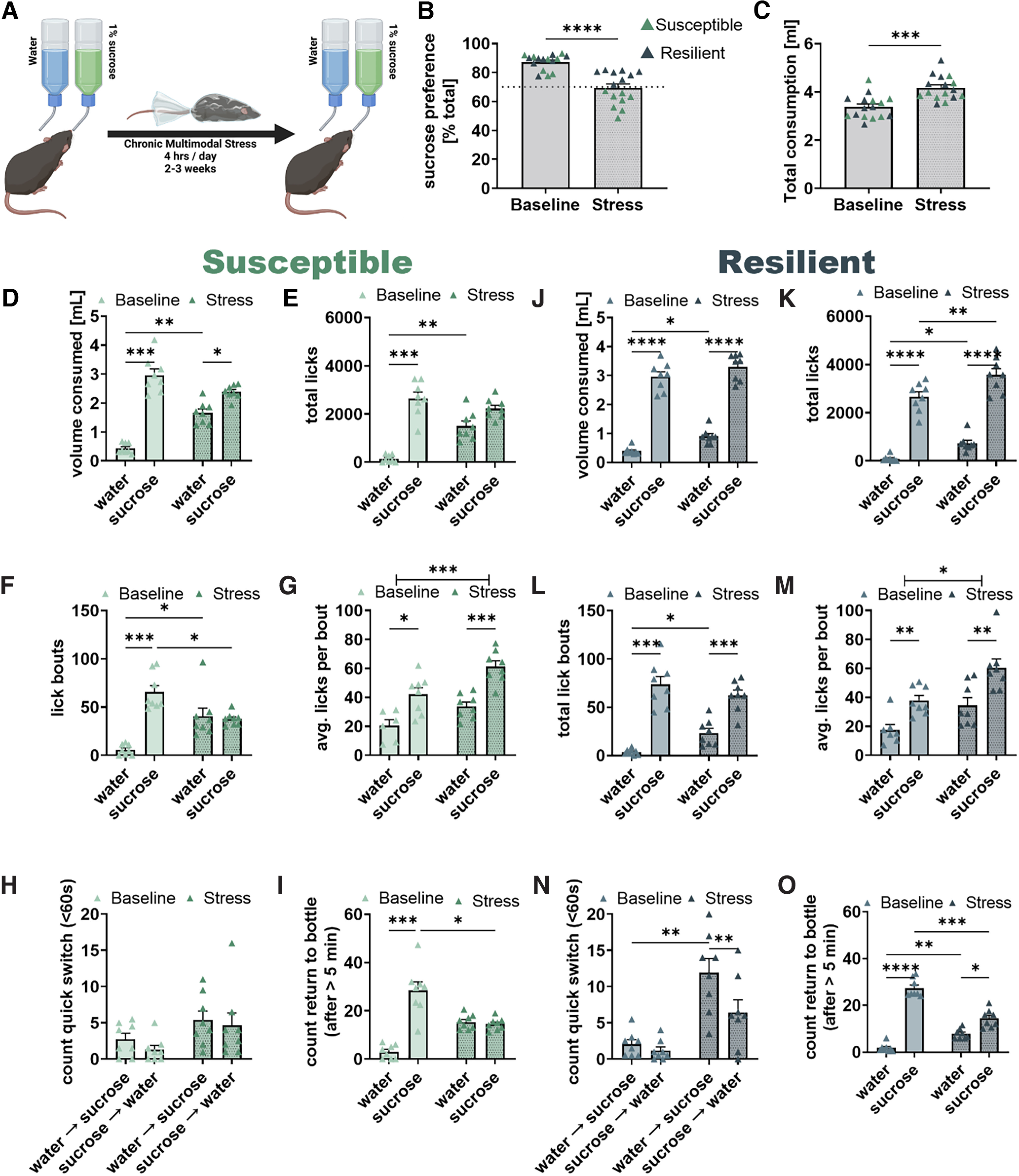
Chronic stress spares hedonic valuation but impairs motivational salience and memory performance in male mice. ***A***, Sucrose preference was measured at baseline and following CMMS in male mice. ***B***, CMMS reduced sucrose preference (***B***, *W* = −134, *p* < 0.0001) and a 70% sucrose preference was used to distinguish stress-susceptible mice from stress-resilient mice. ***C***) CMMS increased total volume consumed (***C***, *t* = 5.145, *p* = 0.0001). ***D–I***, Chronic stress selectively affects drinking behavior in stress susceptible mice. ***D***, Chronic stress affected the preferential consumption of sucrose (*F*_(1,7)_ = 32.22, *p* = 0.0008) by significantly increasing water volume consumed (*t* = 6.95, *p* = 0.0013). However, susceptible mice consumed significantly more sucrose than water both at baseline (***D***, *t* = 9.86, *p* = 0.0001) and after chronic stress (***D***, *t* = 3.99, *p* = 0.031). ***E***, Chronic stress impaired the preferential licking at the sucrose bottle (*F*_(1,7)_ = 15.47, *p* = 0.0057) as susceptible mice licked significantly more at the sucrose bottle than water bottle at baseline (*t* = 8.78, *p* = 0.0003) but not after chronic stress (*t* = 2.46, *p* = 0.24), because of a significant increase in licking at the water bottle (*t* = 5.95, *p* = 0.0034). ***F***, Chronic stress impaired the preferential initiation of lick bouts at the sucrose bottle (*F*_(1,7)_ = 18.72, *p* = 0.0035) as susceptible mice initiated significantly more lick bouts at the sucrose bottle than water bottle at baseline (*t* = 7.47, *p* = 0.0008) but not after chronic stress (*t* = 0.34, *p* > 0.99). This resulted from increased lick bouts at the water bottle (*t* = 3.71, *p* = 0.045) and fewer lick bouts at the sucrose bottle (*t* = 4.03, *p* = 0.03) after chronic stress. ***G***, susceptible mice produced more licks per bout at the sucrose bottle than the water bottle both at baseline (*t* = 3.56, *p* = 0.032) and after chronic stress (*t* = 8.60, *p* = 0.0001). Chronic stress significantly increased licks per bout independent of bottle content (*F*_(1,7)_ = 37.76, *p* = 0.0005). ***H***, Susceptible mice did not exhibit preferential quick-switching either at baseline or following chronic stress (*F*_(1,7)_ = 2.53, *p* = 0.16). ***I***, Stress impaired sucrose location memory performance (*F*_(1,7)_ = 28.94, *p* = 0.0010) as susceptible mice returned more often to the sucrose bottle than water bottle following a pause from drinking at baseline (*t* = 7.36, *p* = 0.0009) but not after chronic stress (*t* = 0.25, *p* > 0.99). Following chronic stress, mice returned more often to the water bottle (*t* = 3.56, *p* = 0.054) and less often to the sucrose bottle (*t* = 4.05, *p* = 0.029). ***J–O***, Chronic stress selectively affects memory performance in stress resilient mice. ***J–M***, Resilient mice consume more sucrose than water (***J***, baseline *t* = 12.98, *p* < 0.0001; stress *t* = 19.12, *p* < 0.0001) and produce more licks (***K***, baseline *t* = 11.14, *p* < 0.0001; stress *t* = 13.74, *p* < 0.0001), lick bouts (***L***, baseline *t* = 8.31, *p* = 0.0004; stress *t* = 7.64, *p* = 0.0007), and licks per bout (***M***, baseline *t* = 5.78, *p* = 0.0024; stress *t* = 5.74, *p* = 0.0014) at the sucrose bottle than water bottle both at baseline and following chronic stress. Chronic stress increases consumption of water (***J***, *t* = 4.36, *p* = 0.02), licks at both water and sucrose bottle (***K***, water *t* = 4.65, *p* = 0.014; sucrose *t* = 5.30, *p* = 0.0067), and lick bouts at the water bottle (***L***, *t* = 4.34, *p* = 0.020) and increased licks per bout independent of bottle content (*F*_(1,7)_ = 10.90, *p* = 0.013). ***N***, Chronic stress significantly affected error correction performance in stress-resilient mice (*F*_(1,7)_ = 27.94, *p* = 0.0011) with mice performing more quick switches from water to sucrose than sucrose to water following chronic stress (*t* = 5.74, *p* = 0.0042) but not at baseline (*t* = 3.33, *p* = 0.074). Chronic stress results in more water-to-sucrose quick-switches (*t* = 5.52, *p* = 0.0053). ***O***, Chronic stress significantly impaired sucrose location memory performance in stress resilient mice (*F*_(1,7)_ = 71.16, *p* < 0.0001). After a pause from drinking, resilient mice directly return to the sucrose bottle more frequently than the water bottle both at baseline (*t* = 14.84, *p* < 0.0001) and following chronic stress (*t* = 3.86, *p* = 0.037). However, chronic stress results both in more returns to the water bottle (*t* = 5.8, *p* = 0.0040) and fewer returns to the sucrose bottle (*t* = 7.38, *p* = 0.0009). *p < 0.05, **p < 0.01, ***p < 0.001, ****p < 0.0001.

**Figure 8.**
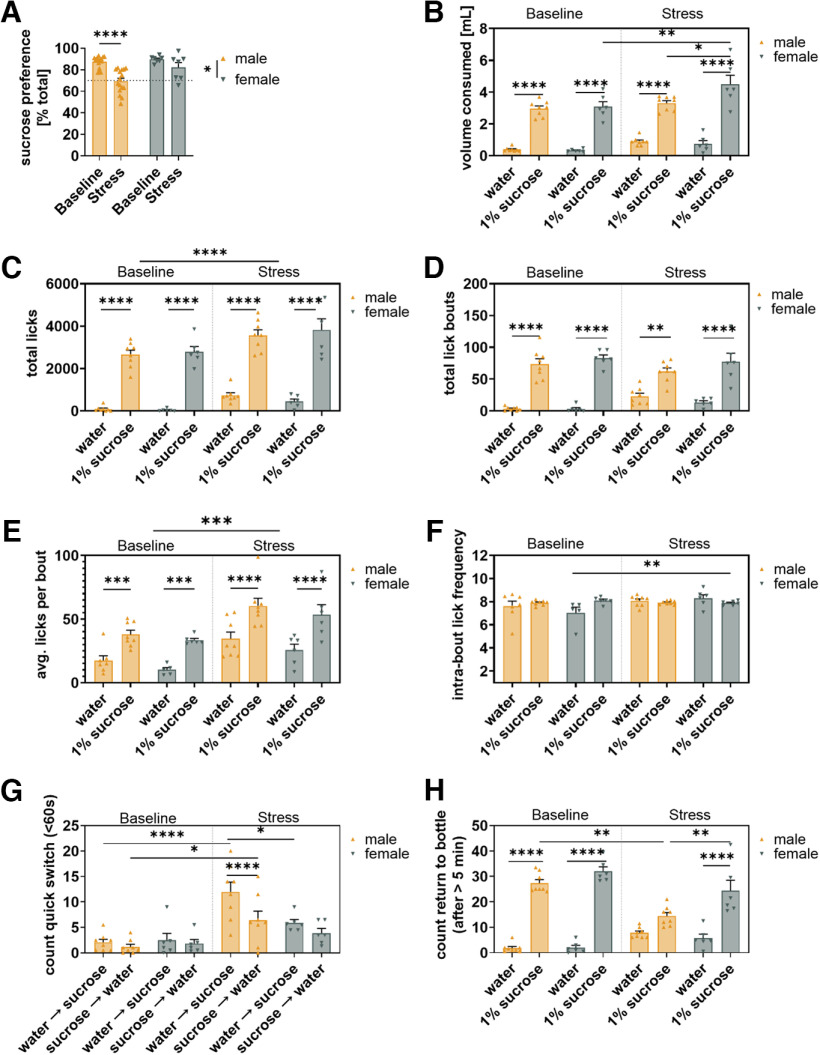
Sex differences in drinking behavior after chronic stress. ***A***, Both stress and sex affected sucrose preference in mice (***A***, stress *F*_(1,21)_ = 24.25, *p* < 0.0001; sex *F*_(1,21)_ = 6.75, *p* = 0.017) with males exhibiting a significant reduction in sucrose preference that was not observed in females (males *t* = 6.28, *p* < 0.0001; females *t* = 1.75, *p* = 0.18). ***B–H***, Comparison of resilient males and resilient females. ***B***, Sex influenced the effect of stress on the differential consumption of water and sucrose (***B***, *F*_(1,12)_ = 6.21, *p* = 0.028) with resilient male and female mice both consuming more sucrose than water at baseline (males *t* = 7.91, *p* < 0.0001; females *t* = 7.36, *p* < 0.0001) and after stress (males *t* = 7.39, *p* < 0.0001; females *t* = 10.00, *p* < 0.0001) but females consuming more sucrose after stress than at baseline (*t* = 5.72, *p* = 0.0011) and more than stressed males (*t* = 3.69, *p* = 0.014). ***C***, Stress affected total licking independently of sex or sucrose (***C***, *F*_(1,12)_ = 37.19, *p* < 0.0001) with resilient males and females producing more licks at the sucrose bottle than at the water bottle at baseline (males *t* = 8.36, *p* < 0.0001; females *t* = 7.77, *p* < 0.0001) and after stress (males *t* = 9.24, *p* < 0.0001; females *t* = 9.53, *p* < 0.0001). ***D***, Stress affected the differential number of lick bouts produced at the water and sucrose bottle (***D***, *F*_(1,12)_ = 12.11, *p* = 0.0045) but resilient males and females produced more lick bouts at the sucrose bottle than water bottle at baseline (males *t* = 8.21, *p* < 0.0001; females *t* = 8.08, *p* < 0.0001) and after stress (males *t* = 4.53, *p* = 0.0016; females *t* = 6.499, *p* < 0.0001). ***E***, Stress affected licks per bout independently of sex or sucrose (***E***, *F*_(1,12)_ = 19.12, *p* = 0.0009) and resilient males and females produced more licks per bout at the sucrose bottle than at the water bottle at baseline (males *t* = 4.78, *p* = 0.0004; females *t* = 4.65, *p* = 0.0005) and after stress (males *t* = 6.32, *p* < 0.0001; females *t* = 5.86, *p* < 0.0001). ***F***, Stress differentially affected intrabout lick frequency at the water and sucrose bottle driven specifically by an increase in intrabout lick frequency at the water bottle in female mice (***F***, *F*_(1,10)_ = 16.20, *p* = 0.0024, *t* = 4.47, *p* = 0.023). ***G***, Sex influenced how stress affected quick switching (<60 s) between water and sucrose bottle (***G***, *F*_(1,12)_ = 7.03, *p* = 0.021) with only stressed male mice exhibiting more quick switches from water to sucrose than sucrose to water (*t* = 7.59, *p* < 0.0001). ***H***, Both sex and stress affected memory performance as measured by returns to sucrose bottle after a pause of >5 min (stress *F*_(1,12)_ = 31.95, *p* = 0.0001; sex *F*_(1,12)_ = 7.34, *p* = 0.019) with resilient males and females returning more to sucrose than water at baseline (males *t* = 9.64, *p* < 0.0001; females *t* = 9.796, *p* < 0.0001) but only females returning more to sucrose than water after stress (*t* = 6.095, *p* < 0.0001). *p < 0.05, **p < 0.01, ***p < 0.001, ****p < 0.0001.

The first experiment sought to investigate through which components of drinking behavior mice expressed a preference for sucrose. A total of 20 male mice were used for this initial experiment and data were analyzed using paired *t* tests or Wilcoxon’s signed rank test for each behavioral component. Represented results are an average of data from two consecutive nights of testing. Statistical details for lick and lick bout data can be found in the legend of Figure 2. Changes in drinking behavior over the course of the test session were described by means and standard error in Figure 3. Finally, memory performance was evaluated and statistical details for these tests can be found in Figure 4. The second experiment further explored the effects of sex as well as the influence of caloric intake and acute stress on the behavioral components underlying the sucrose preference. Eight male mice and seven female mice were first tested in a standard sucrose preference test and a two-way repeated measures ANOVA was performed for each behavioral component using sex and bottle content as factors each with two levels (male-female and water-sucrose, respectively). Statistical details are found in legend to Figure 5. Subsequently mice were tested on the sucrose preference test with various manipulations as described above. An initial three-way ANOVA was performed for each behavioral component to determine whether the sex of the mice affected how each experimental manipulation modulated behavior. While licks per bout at the sucrose bottle was affected by the sex of the mouse as described in the legend to Figure 6, we found no evidence of sex affecting modulation of behavioral components by different experimental manipulations. As such, data were collapsed across sexes and a two-way repeated measures ANOVA was performed for each behavioral component using test context and bottle content as factors with five and two levels, respectively. Statistical details can be found in the legend to Figure 6. Additionally, a two-way repeated measures ANOVA was performed individually for male and female mice to test sex-specific effects again using test context and bottle content as factors with five and two levels, respectively. Statistical details can be found in Extended Data Figure 6-1 for male mice and Extended Data Figure 6-2 for female mice. The third experiment sought to determine which behavioral components were affected by chronic stress resulting in a loss of sucrose preference. Sixteen male and seven female mice were tested before and after chronic stress. Mice were subsequently divided into stress susceptible and resilient mice as described above. While the experiment was initially designed to determine whether sex of the mouse affected the outcomes of chronic stress, we found that female mice were less sensitive to chronic stress in this experiment. Instead, we performed individual analyses on male resilient and susceptible mice and on male and female resilient mice. We performed a two-way ANOVA for each behavioral component using stress and bottle content as factors each with two levels. This analysis was performed individually for the eight stress-susceptible and eight stress-resilient male mice. Statistical details can be found in the legend of Figure 7. We further analyzed the data for the eight male and six female stress-resilient mice using a three-way ANOVA for each behavioral component with sex, stress, and bottle content as factors each with two levels. Statistical details can be found in the legend of Figure 8. All statistical details have been compiled in Extended Data 1.

10.1523/ENEURO.0195-23.2023.f2-1 Extended Data Figure 2-1 Bodyweight was greater for male than female mice throughout the experiments. In cohort 1, male mice weighed 23.13 ± 1.944 g (mean ± SD). In cohort 2, male mice weighed 24.148 ± 2.265 g at the beginning of testing and 26.585 ± 2.391 g at the end whereas female mice weighed only 18.436 ± 1.465 g at beginning of testing and 20.303 ± 0.916 g at the end of testing (mean ± SD). In cohort 3, male mice increased bodyweight up to 24.705 ± 1.717 g before stress whereas females increased weight up to 19.260 ± 1.297 g (denoted by dotted lines). CMMS dampened further increases in bodyweight and ended with a bodyweight of 25.029 ± 1.461 g for males and 19.523 ± 1.054 g for females. Download Figure 2-1, TIFF file.

10.1523/ENEURO.0195-23.2023.f6-1 Extended Data Figure 6-1 Noncaloric sweetener preferential consumption and acute stress impairs memory performance in male mice. ***A***, Following a standard SPT, male mice underwent three preference tests to measure effects of caloric content and acute stress on drinking behavior. First, 1% sucrose was replaced with 0.1% noncaloric sucralose. Second, sucrose preference was tested on nights following acute multimodal stress. Third, sucrose preference was tested while food was restricted to approximately 50% of normal consumption. Finally, mice were re-tested on the standard SPT. ***B***, Mice exhibited an increased preference for 0.1% sucralose than 1% sucrose over water (***B***, Friedman test *p* = 0.0041, *Z* = 2.874, *p* = 0.016) but neither acutes stress nor food-restriction affected sucrose preference. ***C***, ***D***, Experimental manipulations also affected the ratio of sucrose versus water drinking as seen in volume consumed (***C***, *F*_(2.736,17.78)_ = 10.46, *p* = 0.0004) and total licks (***D***, *F*_(2.662,17.30)_ = 6.038, *p* = 0.0065) with mice exhibiting more licks at the sweet bottle when sucrose was replaced with sucralose (***D***, *t* = 4.63, *p* = 0.014). ***E***, Experimental manipulations also affected the number of lick bouts mice initiated at the water or sweet bottle (***E***, *F*_(2.068,13.44)_ = 9.556, *p* < 0.0025) although a *post hoc* analysis found only a trending increase in lick bouts at the sweet bottle when replacing sucrose with sucralose (***E***, *t* = 3.44, *p* = 0.054). ***F***, Male mice consistently exhibited more licks per bout at the sweet bottle compared to the water bottle (*F*_(1,7)_ = 57.92, *p* = 0.0001), which was not found to be significantly affect by experimental manipulations (*F*_(1.817,10.45)_ = 2.57, *p* = 0.13). ***G***, Across all experimental manipulations, mice performed more quick switches (<60 s) from water to sucrose than sucrose to water (***G***, *F*_(1,7)_ = 14.20, *p* = 0.0070). ***H***, Experimental manipulations affected memory performance (***H***, *F*_(2.416,15.70)_ = 7.235, *p* = 0.0043) where replacing sucrose with sucralose resulted in reduced returns to water following a >5-min pause (***H***, *t* = 3.97, *p* = 0.029), and acute stress reduced returns to the sucrose bottle (***H***, *t* = 3.34, *p* = 0.049). Download Figure 6-1, TIFF file.

10.1523/ENEURO.0195-23.2023.f6-2 Extended Data Figure 6-2 Noncaloric sweetener increases preferential consumption and acute stress impairs memory performance in female mice. ***A***, Following a standard SPT, female mice underwent three preference tests to measure effects of caloric content and acute stress on drinking behavior. First, 1% sucrose was replaced with 0.1% noncaloric sucralose. Second, sucrose preference was tested on nights following acute multimodal stress. Third, sucrose preference was tested while food was restricted to approximately 50% of normal consumption. Finally, mice were re-tested on the standard SPT. ***B***, Mice exhibited an increased preference for 0.1% sucralose than 1% sucrose over water (***B***, Friedman test *p* = 0.0045, *Z* = 2.70, *p* = 0.027) but neither acutes stress nor food-restriction affected sucrose preference. ***C***, ***D***, Experimental manipulations also affected the ratio of sucrose versus water drinking as seen in volume consumed (***C***, *F*_(2.206,13.24)_ = 19.66, *p* < 0.0001) and total licks (***D***, *F*_(1.891,11.34)_ = 6.277, *p* = 0.016) with mice consuming more sweetener and when sucrose is replaced with sucralose (***C***, *t* = 4.12, *p* = 0.025) and exhibiting more licks at the water bottle upon retesting under standard conditions (***D***, *t* = 3.93, *p* = 0.030). ***E***, Experimental manipulations also affected the number of lick bouts mice initiated at the water or sweet bottle (***E***, *F*_(2.574,15.44)_ = 13.42, *p* = 0.0002) although a *post hoc* analysis found no significant differences in lick bouts produced across the experimental manipulations. ***F***, Mice consistently produced more licks per bout at the sweet bottle compared to the water bottle (*F*_(1,6)_ = 105.6, *p* < 0.0001) and this was not significantly affected by experimental manipulation (*F*_(1.979,11.38)_ = 2.414, *p* = 0.13). ***G***, Across all experimental manipulations, mice performed more quick switches (<60 s) from water to sucrose than sucrose to water (***G***, *F*_(1,6)_ = 24.12, *p* = 0.0027). H) Experimental manipulations affected memory performance (***H***, *F*_(2.240,13.44)_ = 7.35, *p* = 0.0059) where acute stress reduced returns to the sucrose bottle (***H***, *t* = 4.32, *p* = 0.0197). Download Figure 6-2, TIFF file.

10.1523/ENEURO.0195-23.2023.f6-3 Extended Data Figure 6-3 Circadian pattern of drinking is more robust in males than females and is disrupted by acute stress. Pattern of drinking during standard sucrose preference (***A***, ***B***), when sucrose is replaced with sucralose (***C***, ***D***), after mice have been subjected to acute stress (***E***, ***F***), while mice are undergoing mild food restriction (***G***, ***H***), and upon retesting under standard conditions (***I***, ***J***) in male (left column) and female (right column) mice. Download Figure 6-3, TIFF file.

10.1523/ENEURO.0195-23.2023.ed1 Extended Data 1 Detailed ANOVA and pairwise comparison results. Each sheet within the file corresponds to individual figures and extended data within the manuscript where statistical comparisons were performed. Within each sheet statistical results are divided by panel. Download Extended Data 1, XLS file.

### Statistics

Data were prepared in Excel and statistical tests were performed using GraphPad Prism. Figures were made in GraphPad Prism and MATLAB. Pearson correlations were used to evaluate whether lickometers sufficiently detected drinking behavior. Normality was tested using the Anderson–Darling test. For comparisons of two groups when normality was violated, the Mann–Whitney *U* test was used for unpaired data and the Wilcoxon test was used for paired data, otherwise a *t* test (unpaired or paired) was used. For data containing one dimension of several factors, a Friedman test was used with a Dunn’s multiple comparison *post hoc* test. For comparisons in two or three dimensions, a two-way or three-way repeated measures ANOVA or mixed-effects analysis was used and *post hoc* comparisons were corrected for multiple comparisons with a Šídák’s correction.

### Sex as a biological variable

To determine whether the sex of the mice influenced drinking behavior during the SPT, we used both male and female C57Bl/6 mice. In cases where the sexes have been combined, a prior comparison revealed that there was no significant difference between sexes. Further, in figures where the sexes of the mice have been combined, symbol shapes can be used to identify individual values from female mice (downward-pointing triangle) and male mice (upward-pointing triangle). Male and female mice were housed in separate, but adjacent, rooms and females were tested 1 d after the males.

### Code/software

All MATLAB analysis scripts and 3D designs are available on GitHub (https://github.com/AndreasBWulff/SPT_Lickometry and https://github.com/AndreasBWulff/RestraintTube)

## Results

### How is sucrose preference expressed in stress-naive male mice?

What aspects of drinking behavior account for a preference in stress-naive mice? Twenty male C57Bl/6 mice were singly housed and underwent the SPT as described above while drinking behavior was recorded (Fig. 2*A*).

When only water was present in the home-cage, mice exhibited a preference for one bottle as observed in the volume consumed (Fig. 2*C*, *p* < 0.0001). Mice produced more licks (Fig. 2*D*, *p* < 0.0001) and lick bouts (Fig. 2*E*, *p* < 0.0001) at the preferred water bottle. This indicates that when mice have a preference for one bottle over the other, even without sucrose available, preference is expressed, in part, as a greater number of lick bouts at the preferred bottle. Mice exhibited a strong preference for the 1% sucrose over two nights (Fig. 2*H*, *p* < 0.0001). As with the side preference, mice expressed their sucrose preference by licking more at the sucrose bottle (Fig. 2*I*, *p* < 0.0001) and more lick bouts at the sucrose bottle (Fig. 2*J*, *p* < 0.0001). Interestingly, their total fluid consumption was comparable under both conditions, indicating that they regulate their total intake, regardless of the presence of the sucrose reward (Fig. 2*B*). Mice thus appear to be motivated to seek out the sucrose bottle when they initiate drinking. Because the preference remains when the bottle location is switched, preference is not an artifact of ease of access or habitual approach to the bottles.

We next analyzed drinking behavior within a bout. No difference was seen in the average number of licks within a bout between bottles when sucrose is absent (Fig. 2*F*), despite the presence of a side preference. When sucrose was present, in contrast, mice exhibited significantly more licks per bout at the sucrose bottle than the water bottle (Fig. 2*K*, *p* < 0.0001). More licks per bout for sucrose solution suggests that the mice receive an acute reward feedback while they are drinking, presumably gustatory, that causes them to continue their drinking for a longer time. This rewarding feedback is absent in the case of a side preference.

There was no difference in the frequency of licks during lick bouts in the presence or absence of sucrose (Fig. 2*G*,*L*), indicating that the acute reward feedback does not affect licking motor function.

The SPT is usually performed over two nights with the sucrose bottle location switched between night 1 and night 2. However, some studies test only one night, switching the location of the bottle midway during the night and others test for only 1–2 h. We thus analyzed the time course of drinking behavior in the SPT during the night (Fig. 3). When the lights turn off, we observed a sharp increase in drinking, which peaked about 1 h later, followed by a slow decline. A second spike in drinking was observed around 8 h after the first peak, 2–3 h before lights on. Although mice exhibited a preference for sucrose during the entirety of the session, the difference between the drinking at the sucrose and water bottle was somewhat weaker in the beginning of the session and during periods of sparse drinking. This difference was more pronounced on night 2 than night 1. This indicates that mice can distinguish between the two bottles in <2 h, but that they continue to learn the presence and location of the sucrose bottle and improve their performance over the next 4–5 h. They also identify the new location of the sucrose bottle quickly on night 2.

### A memory component to the expression of sucrose preference

The development of sucrose preference within and between nights suggests that learning and memory may be involved in the expression of sucrose preference. To examine this, we used two different measurements. First, we measured how often mice switched from drinking from one bottle to the other within a 60-s window. We surmised that if mice were aware of the presence of sucrose but did not remember the location of the bottle, then they would realize the error on drinking from the water bottle and quickly switch to the sucrose bottle (Fig. 4*A*). If so, then we predict that there should be more quick switches from water to sucrose than from sucrose to water. Indeed, we found that mice exhibited more quick switches from water to sucrose than vice versa (Fig. 4*B*, *p* < 0.01), although the difference was small. We further found that this difference in quick switches was not present on the first night of testing (Fig. 4*H*) but emerged on the second night of testing (Fig. 4*J*), perhaps as the mice approach the location where the sucrose was located on the night prior and realize their error. However, there was no significant difference in the number of quick switches during the first two and last 2 h of testing (Fig. 4*D*,*F*).

We next measured the number of times mice returned to either the sucrose or water bottle following a >5-min pause from drinking. If mice remember the location of the sucrose solution, then we predicted that they would be more likely to return directly to the sucrose bottle when they start drinking again (Fig. 4*A*). Indeed, we found that mice were significantly more likely to return to the sucrose bottle than the water bottle following a >5-min pause (Fig. 4*C*, *p* < 0.0001), consistent with the contribution of some working memory of the sucrose location. This difference was observed on night 1 (Fig. 4*I*, *p* < 0.05) and more strongly pronounced on night 2 (Fig. 4*K*, *p* < 0.0001). No significant difference in returns to the sucrose or water bottles was observed during the first 2 h of testing (Fig. 4*E*), but mice returned significantly more often to the sucrose bottle during the last 2 h of testing (Fig. 4*G*). This suggests that the memory of the sucrose location develops during the testing session.

Together these data indicate that in stress-naive mice, sucrose preference is expressed through three factors: (1) reward seeking, resulting in more drinking bouts from the sucrose solution; (2) an acute reward feedback, causing longer drinking bouts of the sucrose solution; and (3) a working memory of the location of sucrose bottle.

### The influence of sex, caloric content, and acute stress on sucrose preference

Drinking behavior may be influenced by a variety of other environmental and biological factors. We next investigated how drinking behavior is affected by the sex of the mouse, caloric intake, and acute stress.

We first compared male and female mice on the standard SPT. Male and female mice produced licks that can be divided using the same ILI criteria but a slight shift of the peak toward shorter intervals was observed for the lick pauses in females (Fig. 1). We did, however, not observe any sex difference in the sucrose preference and both male and female mice showed a strong preference for the sucrose solution in volume consumed (Fig. 5*A*, *p* < 0.0001) and total licks (Fig. 5*B*, *p* < 0.0001). Both male and female mice initiated more lick bouts at the sucrose bottle than the water bottle (Fig. 5*C*, *p* < 0.001) and both exhibit more licks per bout at the sucrose bottle than water bottle (Fig. 5*D*, *p* < 0.01). There was also no sex difference in the intrabout lick frequency (data not shown). These data suggest that male and female mice express their sucrose preference via similar drinking behaviors. It should be noted that the females were smaller than the males (Extended Data Fig. 2-1) and thus consume more sucrose per bodyweight than males.

We next examined whether male and female mice exhibited comparable performance on memory measurements. A two-way ANOVA found that mice performed significantly more quick switches from water to sucrose than sucrose to water, largely resulting from the performance of male mice (Fig. 5*E*, *p* < 0.01), although we did not observe significant sex or sex-switch interaction effects. We also found that both male and female mice returned significantly more to the sucrose bottle than water bottle (Fig. 5*F*, *p* < 0.0001) but a two-way ANOVA also revealed a significant sex-bottle interaction which a *post hoc* test revealed to be caused by the females returning more often to the sucrose bottle than the male mice (Fig. 5*F*, *p* < 0.05). During the first 2 h of testing, we further observed a trending sex-bottle interaction (*p* = 0.058). A *post hoc* test revealed that female mice returned significantly more to the sucrose bottle than the water bottle (Fig. 5*G*, *p* < 0.001), which was not observed in the males. However, during the last 2 h of testing both male and female mice returned more to the sucrose bottle than the water bottle (Fig. 5*H*, *p* < 0.0001), with no effect of sex being observed. Thus, while male and female mice both form a working memory of the sucrose location, these data indicate that female mice may develop this memory faster than the male mice.

Caloric intake is known to influence drinking behavior, with postingestive inhibition being known to influence the number of lick bouts at the sucrose bottle (Davis, 1973; Yang et al., 2020). Further, while acute stress does not affect sucrose preference, it may still change the behaviors underlying that preference. To investigate how caloric intake and stress affect drinking behavior, after the standard SPT, we intermittently performed versions of the SPT that were manipulated to (1) replace the 1% sucrose with 0.1% sucralose, a noncaloric sweet tastant, (2) subject the mice to acute multimodal stress on the day before the SPT, and (3) subject the mice to a mild food restriction during the SPT (Fig. 6*A*). Following these manipulated tests, we repeated a standard SPT to determine whether there were any longer-term changes to drinking behavior over time. We observed a significant increase in the expressed preference for the sweetener over water when 1% sucrose was replaced with 0.1% sucralose (Fig. 6*B*, *p* < 0.001; Fig. 6*C*, *p* < 0.01). This resulted from more licks and more lick bouts at the sucralose bottle (Fig. 6*D*, *p* < 0.01; Fig. 6*E*, *p* < 0.01), but no difference in licks per bout (Fig. 6*F*). In contrast, acute stress and mild food restriction did not significantly affect sucrose preference (Fig. 6*B*), although the was a small, but significant decrease in consumed sucrose (Fig. 6*C*, *p* < 0.01) and number of lick bouts at the sucrose bottle (Fig. 6*E*, *p* < 0.01). Interestingly, acute stress also increased licks per bout at the sucrose bottle (Fig. 6*F*, *p* < 0.05).

We also confirmed that mice were significantly more likely to switch from the water to the sweet bottle than vice versa (Fig. 6*G*, *p* < 0.0001) and this was independent of the context of the test. However, we found that replacing sucrose with sucralose resulted in more returns to the sweet bottle and fewer returns to the water bottle following a >5-min pause from drinking (Fig. 6*H*, *p* < 0.05) indicating that mice developed a stronger working memory of the location of the sucralose bottle than of the sucrose bottle. Additionally, acute stress significantly decreased the number of returns to the sucrose bottle (*p* < 0.001) while not affecting the number of returns to the water bottle suggesting that stress may acutely impair working reward location memory. However, after acute stress, mice still returned significantly more to the sucrose bottle than the water bottle indicating that working reward location memory was not entirely abolished.

Male and female mice exhibited similar drinking behaviors and were affected similarly by metabolic changes and acute stress and were for these reasons combined for the analyses described above (Fig. 6). However, sex-specific analyses can be found in extended data figures for male (Extended Data Fig. 6-1) and female (Extended Data Fig. 6-2) mice.

Both male and female mice exhibited a similar pattern of drinking over night with a spike in drinking toward the beginning and the end of the night (Extended Data Fig. 6-3). However, this appeared to be more pronounced in the males than the females (Extended Data Fig. 6-3*C*,*D*). Replacing sucrose with sucralose did not affect the drinking pattern appreciably (Extended Data Fig. 6-3*E*,*F*) but acute stress resulted in increased drinking at the beginning of the night, compared with unstressed mice and compared with the end of the night (Extended Data Fig. 6-3*G*,*H*). This front-loading of drinking was also observed when mice were food-restricted (Extended Data Fig. 6-3*I*,*J*) but is not seen when retested on the standard SPT (Extended Data Fig. 6-3*K*,*L*). The dip in drinking observed around 3 A.M. was also more pronounced in the food-restricted mice, perhaps as they ran out of chow.

Overall, these results indicate that neither caloric content nor acute stress had major effects on sucrose preference.

### Effects of chronic stress on drinking behavior

Since sucrose preference appears to be expressed through several drinking behaviors favoring the sucrose solution, we next set out to determine what aspects of the drinking behavior are affected after chronic stress to result in a loss of sucrose preference.

We tested a third cohort of male and female mice on a standard SPT before and after being subjected to chronic multimodal stress (CMMS; Fig. 7*A*). In this experiment, CMMS significantly reduced sucrose preference (Fig. 7*B*, *p* < 0.0001), and increased total fluid consumption (Fig. 7*C*). There was a significant effect of sex (*p* < 0.05) and a trending sex-stress interaction (*p* = 0.058) on sucrose preference, with female mice somewhat less sensitive to chronic stress than male mice (Fig. 8*A*). Because we wished to determine the changes underlying loss of sucrose preference, we divided mice into stress susceptible or stress resilient based on whether they had <70% or >70% sucrose preference following chronic stress (Hesselgrave et al., 2021; Troppoli et al., 2022). Of 16 male mice, eight were susceptible, and of seven female mice, only one was susceptible. We thus decided to use only male mice for analysis of differences between resilient and susceptible mice to avoid possible interference of sex differences.

Consistent with the loss of sucrose preference, susceptible mice consumed more water following chronic stress than during baseline (Fig. 7*D*, *p* < 0.01). Resilient mice also showed a small, but significant increase in their consumption of water after stress (Fig. 7*J*, *p* < 0.05).

Decreased sucrose preference following chronic stress in susceptible mice resulted from more licks (*p* < 0.01) and lick bouts (*p* < 0.05) at the water bottle and fewer lick bouts at the sucrose bottle (*p* < 0.05) than at baseline (Fig. 7*E*,*F*). Susceptible mice showed significantly more licks per bout at the sucrose bottle compared with the water bottle both before (*p* < 0.05) and after chronic stress (*p* < 0.001) and, while a significant effect of stress was found (*p* < 0.0001), this effect was observed for both bottles (Fig. 7*G*). Neither chronic stress nor bottle content affected the intrabout lick frequency (data not shown). Chronic stress thus increased the number of licks per bout at both the water bottle and sucrose bottle.

Resilient mice increased licks at both the water bottle (Fig. 7*K*, *p* < 0.05) and sucrose bottle (*p* < 0.01) and while they increased the number of lick bouts initiated at the water bottle after stress (Fig. 7*L*) they continued to initiate more lick bouts at the sucrose bottle than water bottle after stress (*p* < 0.001) which was not observed in the stress susceptible mice (Fig. 7*F*). Similar to susceptible mice, resilient mice produced more licks per bout at the sucrose bottle than water bottle both before (Fig. 7*M*, *p* < 0.01) and after stress (*p* < 0.01), with stress increasing licks per bout independently of bottle content (*p* < 0.05).

The biggest effect of chronic stress was on the return to the sucrose bottle. At baseline, both susceptible and resilient mice returned to the sucrose bottle significantly more often than to the water bottle after a pause (Fig. 7*I*,*O*, *p* < 0.0001). After chronic stress, susceptible mice show no difference in number of returns to either bottle (Fig. 7*I*) and, while resilient mice return more to the sucrose bottle than water bottle after stress (Fig. 7*O*, *p* < 0.05), they return significantly more to the water bottle (*p* < 0.01) and significantly less to the sucrose bottle (*p* < 0.001) suggesting some impairment to their reward location memory.

Resilient mice continued to make significantly more quick switches from the water bottle to the sucrose bottle after chronic stress (Fig. 7*N*, *p* < 0.001). At baseline, the susceptible mice did not exhibit a difference in the number of quick switches between the water bottle and the sucrose bottle (Fig. 7*H*,*N*), unlike the previous cohorts, making it impossible to detect an effect of chronic stress.

Taken together, chronic stress reduced the initiation of lick bouts at the sucrose bottle, without affecting the duration of the lick bouts once initiated in susceptible but not resilient male mice. Memory performance was also impaired by chronic stress in both resilient and susceptible mice, as indicated by the failure to return directly to the sucrose bottle. Resilient mice appeared to compensate for this loss of reward location memory by switching quickly from the water bottle to the sucrose bottle, which was not observed in susceptible mice.

We examined sex differences in the drinking behavior following stress in resilient male and female mice before and after chronic stress. Female stress resilient mice exhibited significant increase in sucrose consumption following chronic stress (Fig. 8*B*, *p* < 0.01) which was not observed in male mice resulting in female mice consuming more sucrose than male mice following stress (Fig. 8*B*, *p* < 0.05). However, no sex difference was observed in licks, lick bouts and licks per bout (Fig. 8*C–D*) with both male and female mice exhibiting significant increase in licks and licks per bout across bottles after stress (Fig. 8*C*,*E*, *p* < 0.001). Male resilient mice exhibited more water-to-sucrose than sucrose-to-water quick switches (Fig. 8*G*, *p* < 0.0001) and a loss of preferential return to the sucrose bottle following a >5-min pause from drinking (Fig. 8*H*) after chronic stress which was not observed in female mice.

Thus, female and male resilient mice exhibited comparable reward seeking behavior in their initiation of lick bouts and palatability response in the form of licks per bout. Male resilient mice, however, exhibited impaired reward location memory that was not observed in female mice. Male resilient mice, but not females, compensated for this impairment by switching quickly to the sucrose bottle when erroneously approaching the water bottle first.

## Discussion

We used lickometry to study drinking behavior in mice during the SPT to understand how sucrose preference is expressed and lost following chronic stress.

### Sucrose preference is mediated by reward responsiveness, seeking, and memory

We found that sucrose preference is expressed via three separable behavioral components. We observed an acute reward response to the palatable sucrose solution, evidenced as longer drinking bouts for sucrose than water. The longer drinking bouts suggest rapid positive feedback within the millisecond to second time range of the individual bout. Additionally, mice displayed reward seeking, resulting in a greater number of drinking bouts at the sucrose bottle (Fig. 2).

Mice form memories of specific reward locations (Gauthier and Tank, 2018). We observed a memory component to the SPT in two measures. After training, mice were more likely to approach the sucrose bottle first, showing they remembered its location (Fig. 4). They also updated this location rapidly after bottles were switched. Female mice exhibited better reward location memory performance than males (Fig. 5), with females developing a reward location memory faster than males in the first hours of the SPT (Fig. 5).

We also observed more switches from water to sucrose than vice versa. An increase in switches between bottles may also be reflective of enhanced exploratory behavior of male mice but, if so, then switches should be evenly distributed between the two switch directions (i.e., even number of switches from water to sucrose and from sucrose to water). We observed a greater number of switches from water to sucrose, suggesting that when mice approach the less rewarding water bottle, they remember that the other bottle has sucrose and quickly switch to the sucrose bottle; a form of error correction (Fig. 4). This effect was small, but significant, suggesting that these error corrections were rare. Males exhibited more error correction than females (Fig. 5).

The temporal resolution of lickometry provides a better understanding of the pattern of drinking and the time when sucrose preference is optimally expressed which should be considered when designing experiments. While sucrose preference is evident on the first night, it is only strongly expressed after several hours of testing. Additionally, mice developed a preference for one bottle even when both contained water. Performing the SPT over shorter periods of time, in a single test only, and without switching the location of the bottle may lead to less accurate measurement of sucrose preference.

### Caloric content does not appreciably affect drinking behavior during standard SPT

The behavioral components underlying the SPT could be driven by the sweetness of the solution or its caloric content. Gut-brain signaling can be responsible for a preference for sucrose over artificial sweeteners (Buchanan et al., 2022). Moreover, increasing sucrose concentrations above 3–10% results in a decline in drinking bouts because of postingestive inhibitory feedback. Food restriction increases the number of lick bouts without affecting the number of licks per bout (Davis, 1973; Davis and Smith, 1992; Spector et al., 1998; Yang et al., 2020). On the other hand, pairing noncaloric sweetener with intragastric infusion of carbohydrates results in increased consumption and licks per bout, while reducing number of bouts via postingestive positive feedback (for review, see Sclafani, 2001).

We found that mild food restriction did not affect the number or duration of lick bouts in the SPT (Fig. 6). This may be because of the low concentration of sucrose used (1%), which is less than that observed to induce gastric feedback (Davis and Smith, 1992; Spector et al., 1998; Sclafani, 2001). The food-restriction used in this experiment is very mild assuming that if caloric content influenced consumption during the standard SPT, we should be in a sensitive range where small changes to caloric need could be observed in the drinking behavior. It is likely that a more severe food-restriction would affect drinking behavior. Thus, demand for caloric intake seems to drive sucrose consumption only in a more food-deprived state.

Replacing sucrose with a 10-fold lower concentration of sucralose (Buchanan et al., 2022), revealed a small, but significant, enhancement of preference for sucralose compared with 1% sucrose, expressed as an increase in the number of total licks and lick bouts (Fig. 6). Sucralose substitution also enhanced reward location memory performance, with mice returning less to the water bottle and more to the sucralose bottle (Fig. 6). The enhanced preference and memory performance may be caused by a relief of inhibitory feedback from the gut that would otherwise occur when mice consume sucrose. However, it may also be reflective of an enhanced perceived sweetness of the sucralose solution compared with the sucrose solution. While mice prefer a 20× higher sucrose solution over sucralose (Buchanan et al., 2022), other studies suggest that the preference threshold for sucralose is about 100× higher than that of sucrose when compared with water (Bachmanov et al., 2001). Overall, the relatively small differences between sucrose and sucralose further suggests that caloric intake plays only a minor role in the SPT.

### Stress impairs reward memory and reduces reward seeking resulting in an anhedonic-like phenotype

The SPT is widely used to test for stress-induced anhedonia. We therefore tested how acute and chronic stress affected drinking behavior during the SPT. Interestingly, while acute stress did not significantly affect overall sucrose preference, it did produce a range of effects on drinking behavior. While acute stress reduced the number of lick bouts specifically at the sucrose bottle, it also increased the number of licks per bout at both the sucrose and water bottle (Fig. 6). Acute stress further reduced the number of times mice returned directly to the sucrose bottle, without affecting returns to the water bottle, resulting in an overall reduced reward location memory performance (Fig. 6). Finally, acutely stressed mice drink more at the beginning of the dark phase and less drinking throughout the rest of the night (Extended Data Fig. 6-3). This contrasts with stress-naive mice, which exhibit a smaller spike in drinking during the first few hours of the dark phase and a second spike in drinking of approximately the same size during the last few hours (Fig. 3; Extended Data Fig. 6-3). Perhaps stressed mice seek to rehydrate themselves by initiating a few but long lick bouts in the beginning of the night followed by less drinking while they rest. Interestingly, these results conflict with data from rats, where acute foot-shock stress did not significantly affect cluster size or cluster number but did reduce intracluster lick frequency (Mitra et al., 2016). This discrepancy may be because of species differences, different stressors, different test time (16 vs 1 h), or different sucrose concentrations (1% vs 10%).

Finally, comparison of susceptible and resilient male mice after chronic stress revealed that loss of sucrose preference was because of a reduced number of lick bouts at the sucrose bottle, indicative of impaired reward seeking (Fig. 7). Susceptible mice continued to make more licks per bout at the sucrose bottle compared with the water bottle, suggesting that they still found the sucrose solution palatable. Memory impairments induced by chronic stress may also contribute to the reduction in sucrose preference, but both resilient and susceptible males exhibited impaired performance. The detrimental effects of chronic stress on motivation have been well established using methods such as the forced-ratio or progressive-ratio lever pressing task (Kleen et al., 2006; Hollon et al., 2015). Given the low and equal effort required to drink from the two bottles in the SPT, it might be assumed that loss of sucrose preference is because sucrose is no longer perceived as palatable. Our results suggest, however, that the palatability of sucrose remains intact following chronic stress and that the loss of sucrose preference results from reduced reward seeking.

Female mice were less susceptible to chronic stress than male mice in our experiment (Fig. 8). Producing stress-induced depressive-like behaviors in female mice has proven challenging (Autry et al., 2009; Borrow et al., 2019; Cao et al., 2021), and often requires use of different stressors than for males (Hodes et al., 2015; Harris et al., 2018). Resilient females may be better protected against stress-induced cognitive impairments because, unlike males, they retained reward location memory (Fig. 8). Other rodent studies have also suggested that females are better protected against stress-induced cognitive impairments than males (Luine, 2002; Bowman et al., 2003).

Our results are generally similar to the recent study of Verharen et al. (2023) which used a similar approach, along with computation modeling, to tease apart behavioral components of expressed sucrose preference in naive and stressed states. Similar to our findings, they reported that sucrose preference was expressed through both increased lick bouts (called choices in their paper) and licks per bout, as well as in computed hedonia and learning rate parameters. However, some key differences between their study and ours are worth noting. First, they performed most sucrose preference tests while mice were in a food-deprived state, whereas we did not use food deprivation. Acute food deprivation may influence a wide range of behaviors including hedonic, metabolic, and cognitive performance. When chow was present in their study, sucrose preference was greatly reduced largely because of a reduction in number of lick bouts. Their observation is consistent with other lickometry studies showing that severe food-restriction activates gut-brain circuits to drive sucrose intake (Davis and Smith, 1992; Spector et al., 1998; Sclafani, 2001). Another difference is that Verharen and colleagues modelled the role of learning in sucrose preference, and found that learning was unaffected by chronic stress. In contrast, we used a more direct approach to estimate reward memory by counting returns to the sucrose bottle and observed significant memory impairment after chronic stress. Finally, their study found that chronic mild stress reduced sucrose preference by reducing both number of lick bouts and licks per bout and a general reduction in number of licks. However, here we found that CMMS increased consumption while decreasing lick bouts and memory performance but found no change in licks per bout. We further expanded on this finding by dividing stressed mice into susceptible and resilient and showing that while both susceptible and resilient mice had reduced memory performance, only susceptible mice showed a decrease in number of lick bouts. These discrepancies may be explained by differences in hunger state, differences in test lengths and number of test sessions and/or differences in lick bout detection parameters.

### Behavior-specific effects underlying stress-induced loss of sucrose preference points to anatomic loci of interest for future studies in animal models of depression

The SPT is considered one of the better measures of depression-relevant reward behavior. It holds some construct validity because loss of sucrose preference can be induced by chronic stress, a major risk factor for human depression. It has some face validity as the loss of sucrose preference represents a murine anhedonic-like state, resembling this core symptom of human depression. And it has some predictive validity because selective serotonin reuptake inhibitors are able to restore sucrose preference in chronically stressed mice when administered chronically, but not acutely (LeGates et al., 2018).

One limitation of the SPT is that the brain regions and circuits involved in expressing a preference and how these systems are impaired following chronic stress are not known. This may be a result of the pleiotropy of behavioral components underlying this test. Our results suggest more precise regional targets for the study of stress-induced loss of sucrose preference. The hippocampus encodes the location of rewards (Gauthier and Tank, 2018; Sosa and Giocomo, 2021). Excitatory inputs from hippocampus to nucleus accumbens enhance subjective palatability in the form of increased licks per bout (Yang et al., 2020). Here, we found that reward location memory was impaired in both resilient and susceptible male mice following chronic stress. Reward location memory is supported by hippocampus-nucleus accumbens projections and their synapses onto dopamine D1 receptor expressing neurons are weakened following chronic stress (LeGates et al., 2018), suggesting a possible circuit explanation for changes in memory for the location of the sucrose bottle. Dopamine activating D1 receptors is a key factor in the initiating of lick bouts (for review, see Johnson, 2018). D1 receptor antagonists reduce the number of lick bouts without affecting licks per bout for sucrose (D’Aquila, 2010), similar to the behavior observed here after chronic stress. Additionally, the orbitofrontal cortex contains neurons that predict the initiation of a lick bout and their disruption affects the number of lick bouts initiated (Gutierrez et al., 2006). These systems are possible targets for better understanding the neurobiological mechanisms of stress-induced anhedonia.

## Conclusion

In concussion, our analysis of drinking behavior during the SPT in mice revealed that acute hedonic responsivity, motivational salience, and reward location memory all contribute to the expression of sucrose preference, with little influence of caloric content. Male and female mice exhibited similar drinking behavior, although females exhibited reduced hedonic responsivity and better memory performance than males. Chronic stress did not affect palatability, but impaired location memory. Stress-induced impairment of reward seeking distinguished stress susceptible from stress resilient mice. These results inform our understanding of the behavioral and neurobiological mechanisms underlying stress-induced anhedonic-like behavior as measured using the SPT.

## Synthesis

Reviewing Editor: Erica Glasper, The Ohio State University Decisions are customarily a result of the Reviewing Editor and the peer reviewers coming together and discussing their recommendations until a consensus is reached. When revisions are invited, a fact-based synthesis statement explaining their decision and outlining what is needed to prepare a revision will be listed below. The following reviewer(s) agreed to reveal their identity: Silvana Chiavegatto.

**Overview**: This study replicates and extends some of the finding presented by Verharen et al in a 2023 Nature Communications publication. In this manuscript, the authors used an affordable, and highly accessible home cage lick-o-meter to demonstrate that sucrose preference is expressed via the response to the sucrose palatability and reward seeking as well as sucrose memory in male and female mice. Additionally, both acute and chronic stress reduces reward seeking and sucrose memory. Finally, the authors demonstrate that reduced reward seeking distinguishes stress-susceptible from stress-resilient mice. The lick-o-meters represent a technological advancement field. While the reviewers find merit in the work as is, there remain major concerns that should be addressed in a revised version of this manuscript. Overall, the manuscript should be significantly reorganized (e.g., headings, subheadings) such that the information can flow logically. Moreso, it is important for the authors to clearly state how this manuscript fills the gaps in our knowledge that the Verharen et al publication did not. It is very important for the authors to state what the important new finding of this study are.

**Major Concerns**

Introduction

1. Page 5, Lines 7-10 - This section should be updated to include the Verharen et al 2023 reference as they also reported licking microstructure, etc. in that publication.

Methods

1. Beginning on Page 5 - The animal section should be expanded to include detailed information, including time of lights on and off, temperature, type of chow, cages, vivarium, etc.

2. Page 6, Lines 5-16 - To increase reproducibility, please increase the detail of the lickometry system methods. While based on the system described by Hayar et al., 2006, this system is different. For example, please state whether the same device was reused in each mouse.

3. Page 6, Line 24 - Information pertaining to removal of individual mouse data should be moved to the Results section. Additionally, a general error estimate for each step could be included to provide guidance for care in future studies (e.g., clogs in the plastic valve, leaks, movement of the metal plate, etc.)

4. Page 7, Lines 12-25 - Davis and Smith (1992) was cited to define groups based on inter-lick intervals (ILIs): bursts, clusters, and pauses. It is unclear why the authors did not use this standard nomenclature throughout the manuscript; no explanation was provided. The authors are encouraged to provide an explanation for using a different nomenclature and to highlight the advantages of the new approach they employed. Along these lines, Supplementary Figure 3S, should be included as one of the main figures in the Results section.

5. Page 7, Lines 22-23 - Please move the following to the Results section : “a slight leftward shift of the peak was observed for the lick pauses in females”. The implications of this should be added to the Discussion section.

6. Page 8 - Please state the sweetness equivalence of the sweetener used compared to sucrose?

7. Page 9, Line 1 - Reliability and reproducibility are concerns when it comes to the use of non-scientific grade sugar. The same concern is raised when it comes to the “Trap Shack Company” and “MIDSCI” to describe a tube.

8. Page 9, Line 3 - The authors should describe how and when the 50% of normal food consumption was determined. Also, please state what the reasoning behind the choice of exact 50% reduction was.

9. Page 9, Lines 8-15 - Please describe the multimodal restraint stress protocol, including length of time, and appropriately cite references relating to this method of stress. Additionally, provide more details regarding the acute stress protocol. How many hours before the sucrose test was acute stress applied?

10. Page 9, Lines 13-15 - It is not clear why the authors chose 70% of sucrose preference as the criterion to discriminate between resilient and susceptible mice.

11. Page 9, Lines 19+ - The experimental design is not clear. Independent sets of mice should have been used to explore the effects of sex, and caloric intake/acute stress, on lickometer performance. Multiple manipulations in sequence, as performed, may have masked or confounded interactions, particularly when it comes to motivation and memory to consume sucrose. Also, the N size for the females is low, making it difficult to determine the effects of sex on outcome measures.

Results

1. Page 15, Lines 13-14 - Please add the bodyweight data for males and females.

2. Page 18, Lines 7-8 - Clarity is needed regarding the use of the 70% criterion usage in both males and females. Figure S5 panel A suggests that a higher cut-off would give 4 resilient and 3 susceptible females. It is suggested that using 2 or 3 standard deviations of the % sucrose preference from the control mean as criterion for male or female mice should be used, as done in Alves-dos-Santos et al, Neurobiology of Stress, 2020 - doi.org/10.1016/j.ynstr.2020.100221).

Discussion

1. Page 21, Lines 15-19 - The authors state “We also observed more switches from water to sucrose than vice versa. We suggest that when mice approach the less rewarding water bottle, they remember that the other bottle has sucrose and quickly switch to the sucrose bottle; a form of error correction (Fig. 3). This effect was small, but significant, suggesting that these error corrections were rare. Males exhibited more error correction than females (Fig. 4).” The authors are encouraged to consider another possible interpretation of the data, such that the behavior could reflect enhanced exploratory behavior in male mice.

2. Page 22 - Please clarify. Should the increased number of total licks and lick bouts for 0,1% sucralose compared to 1% sucrose, as well as an enhanced reward location memory performance (Fig. 5), be due to its higher sweetness at this concentration to match with the sucrose?

Figures

1. Figure 1 - Please clarify the discrepancy: 16 male mice are represented at water-water to determine the side preference, but for the sucrose preference (water-sucrose), 20 mice are represented. The text suggests the same mice were used on subsequent days.

2. Figure 2 - Night 1 has 19 rows (mice) and night 2 has 20 rows. Please edit the legend to reflect this difference.

3. Figure 3 - Please clarify. Is A-B-C the average of two nights? If so, please add this information to the legend. Also, please correct the same legend (line 9) to reflect that mice did NOT differ in number of quick switches.

4. Figure 4 - Please mention 1% sucrose.

5. Figure 5 - Outcome measures may be confounded by using mice in sequence (mention above). The authors should represent the water-sucrose and water sucralose for both male and female mice. Also, please check Figure S4 regarding maintaining the circadian pattern of drinking only for panels A-D.

6. Figure 6 - Please state whether these are finding for male mice only.

7. Supplemental Figure S5 should be included as a main figure.

**Minor Concerns**

1. Page5, Line 23 - Please use the correct (C57BL/6J) name of the mouse strain.

2. Page 9, Line 12 - What is meant by ca. 9:00 - 13:00?

3. Page 22, line 14 - should be “sucralose” instead of “sucrose”

4. Page 24 - define “CMMS”

5. Figure 1A - Please correct the side of the 1% sucrose bottle at Night 1. According to the text, it should be placed on the non-preferred side).

6. Figure 2 could be improved by using different colors for the raster plots. The colors should correspond with line graph colors representing the water and sucrose licks.
