# Supplementary figures and images for: How Sucrose Preference Is Gained and Lost: An In-Depth Analysis of Drinking Behavior during the Sucrose Preference Test in Mice
Source: eNeuro. 2023 Sep 26;10(9):ENEURO.0195-23.2023. doi: 10.1523/ENEURO.0195-23.2023 (PMC10540674; doi:10.1523/ENEURO.0195-23.2023)

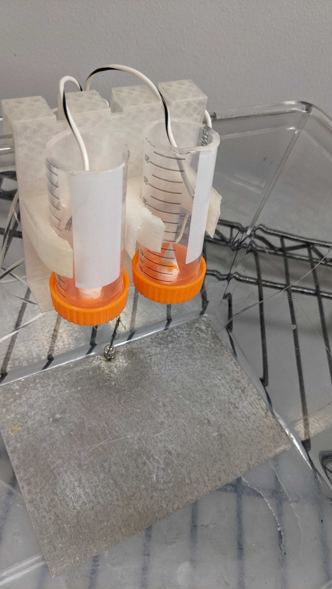

Supplement: Extended Data Figure 1-1 — Lickometry setup. Image depicts the in-cage component of the lickometry setup used to record drinking behavior of mice during the sucrose preference test. Bottles were made from 50-ml falcon tubes with the ends cut off and plastic sippers inserted into the screw lid. A custom-designed 3D-printed bottle holder was used to secured the bottles to the side of the mouse cages in a way that allowed the cages to be completely closed during the testing. An aluminum plate was placed at the bottom of the mouse cages under the bottles with a wire connected to the ground of the digitizer. A wire was connected from the inside of each bottle to the signal inputs of a digitizer. Additionally, cages contained standard bedding not depicted in the image. Download Figure 1-1, TIFF file. [file enu-eN-NWR-0195-23-s03.tif]

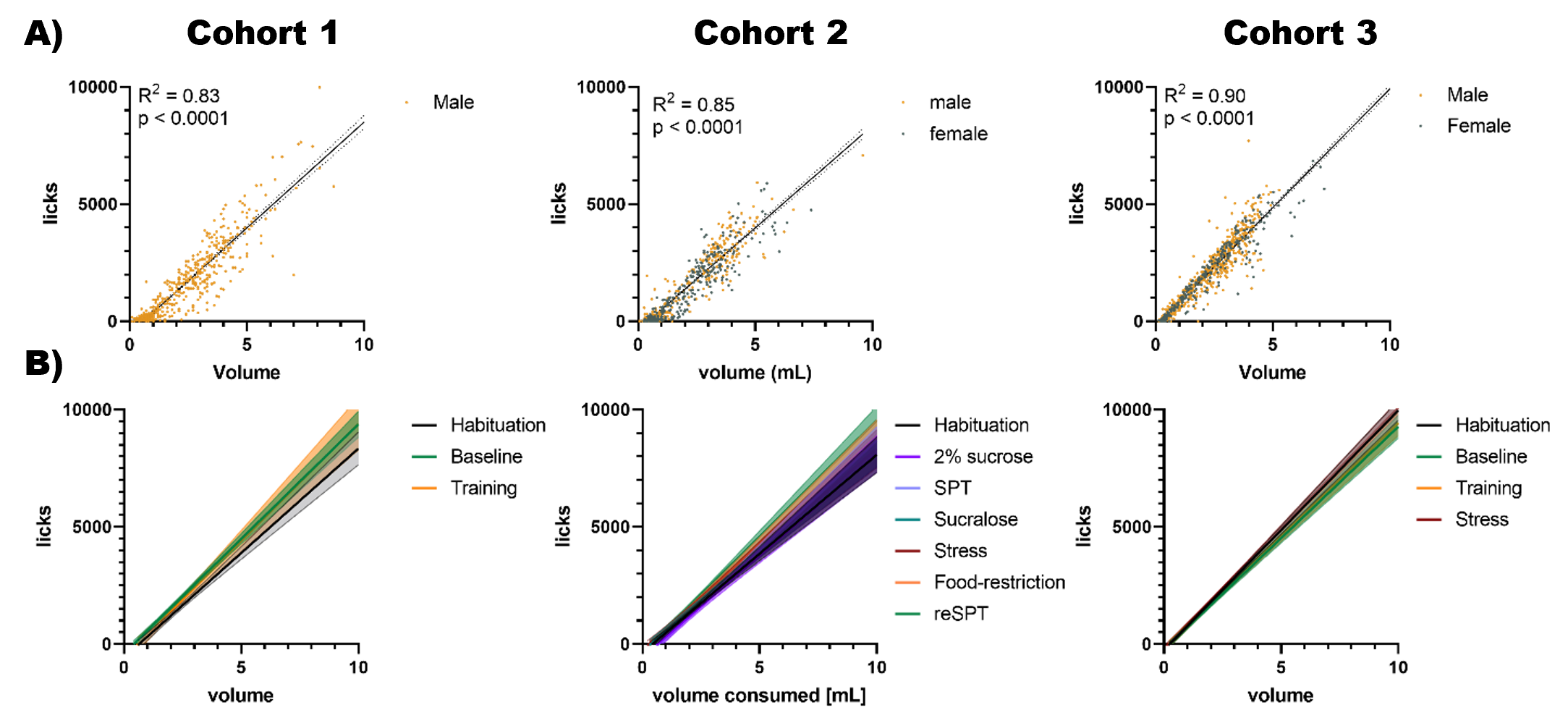

Supplement: Extended Data Figure 1-2 — Capacitive lickometer accurately captures the drinking behavior of mice during the sucrose preference across sex and behavioral setting. Correlations between total licks and volume consumed were performed for all included recording sessions. A, In all cohorts, this correlation was highly significant (p < 0.0001) with an R2 = 0.83 in cohort 1, R2 = 0.85 in cohort 2, and R2 = 0.90 in cohort 3. B, The slope of this correlation was not significantly different between different experimental sessions either for (D) cohort 1 (F(2,228) = 1.55, p = 0.21), (E) cohort 2 (F(7,578) = 1.890, p = 0.07), or (F) cohort 3 (F(3,656) = 2.041, p = 0.11). Download Figure 1-2, TIFF file. [file enu-eN-NWR-0195-23-s04.tif]

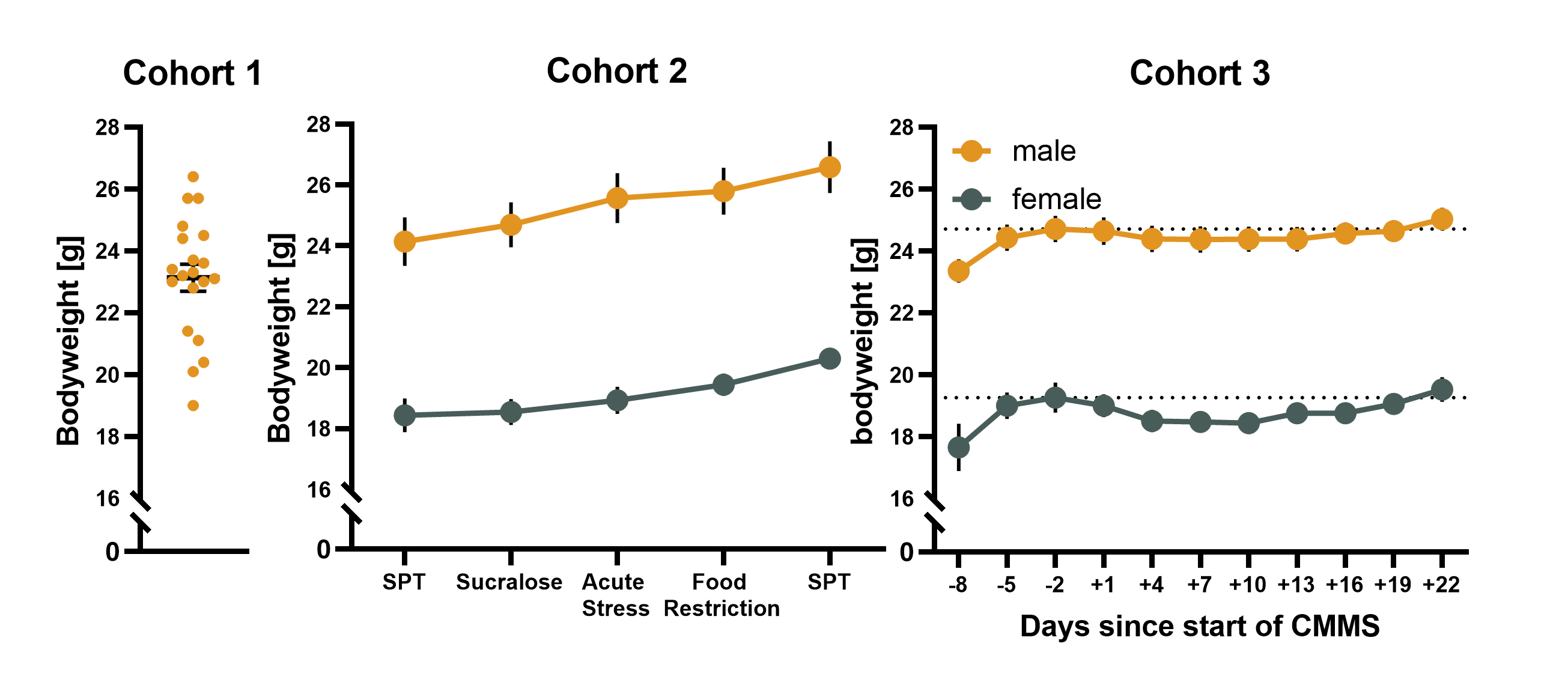

Supplement: Extended Data Figure 2-1 — Bodyweight was greater for male than female mice throughout the experiments. In cohort 1, male mice weighed 23.13 ± 1.944 g (mean ± SD). In cohort 2, male mice weighed 24.148 ± 2.265 g at the beginning of testing and 26.585 ± 2.391 g at the end whereas female mice weighed only 18.436 ± 1.465 g at beginning of testing and 20.303 ± 0.916 g at the end of testing (mean ± SD). In cohort 3, male mice increased bodyweight up to 24.705 ± 1.717 g before stress whereas females increased weight up to 19.260 ± 1.297 g (denoted by dotted lines). CMMS dampened further increases in bodyweight and ended with a bodyweight of 25.029 ± 1.461 g for males and 19.523 ± 1.054 g for females. Download Figure 2-1, TIFF file. [file enu-eN-NWR-0195-23-s05.tif]

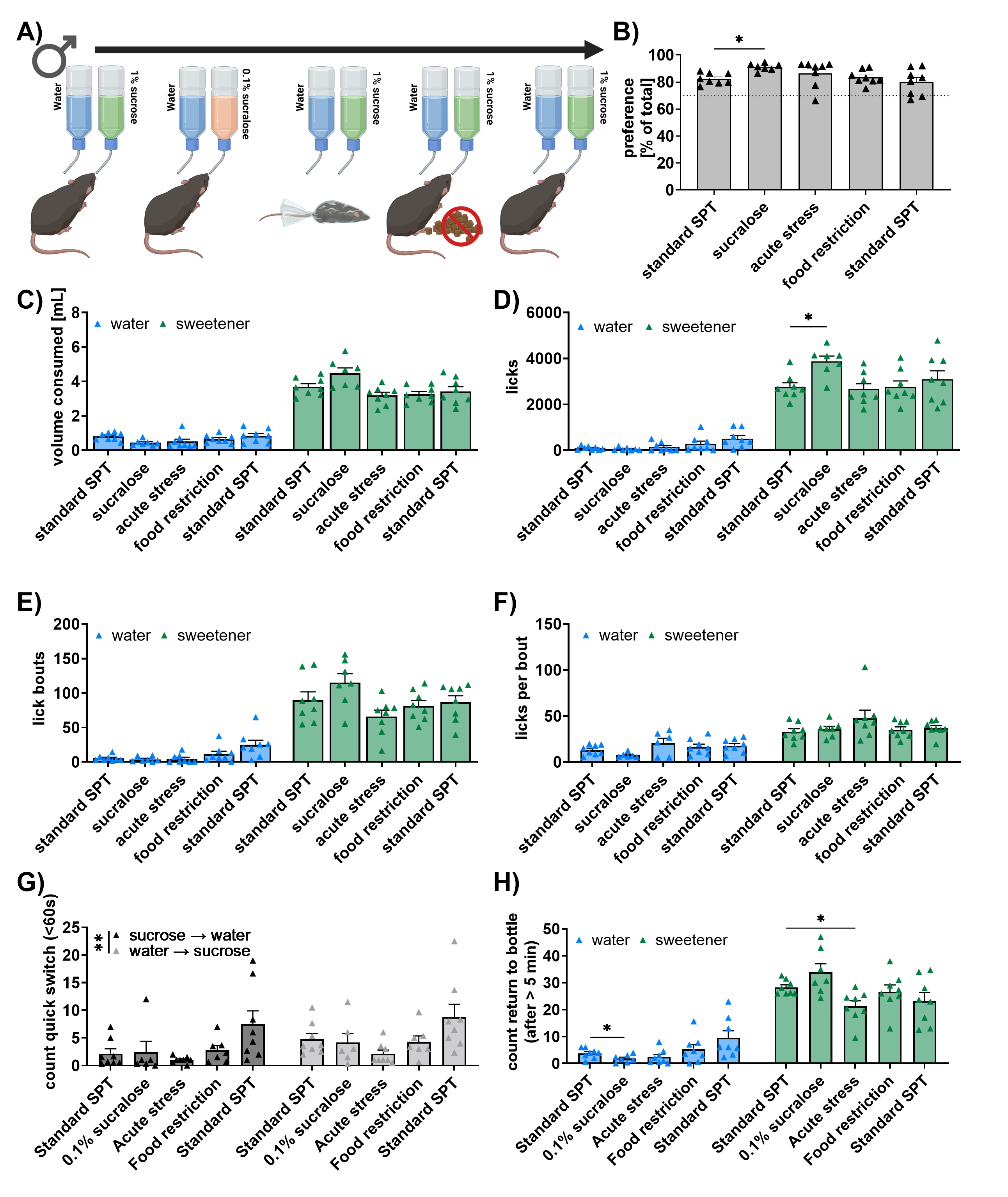

Supplement: Extended Data Figure 6-1 — Noncaloric sweetener preferential consumption and acute stress impairs memory performance in male mice. A, Following a standard SPT, male mice underwent three preference tests to measure effects of caloric content and acute stress on drinking behavior. First, 1% sucrose was replaced with 0.1% noncaloric sucralose. Second, sucrose preference was tested on nights following acute multimodal stress. Third, sucrose preference was tested while food was restricted to approximately 50% of normal consumption. Finally, mice were re-tested on the standard SPT. B, Mice exhibited an increased preference for 0.1% sucralose than 1% sucrose over water (B, Friedman test p = 0.0041, Z = 2.874, p = 0.016) but neither acutes stress nor food-restriction affected sucrose preference. C, D, Experimental manipulations also affected the ratio of sucrose versus water drinking as seen in volume consumed (C, F(2.736,17.78) = 10.46, p = 0.0004) and total licks (D, F(2.662,17.30) = 6.038, p = 0.0065) with mice exhibiting more licks at the sweet bottle when sucrose was replaced with sucralose (D, t = 4.63, p = 0.014). E, Experimental manipulations also affected the number of lick bouts mice initiated at the water or sweet bottle (E, F(2.068,13.44) = 9.556, p < 0.0025) although a post hoc analysis found only a trending increase in lick bouts at the sweet bottle when replacing sucrose with sucralose (E, t = 3.44, p = 0.054). F, Male mice consistently exhibited more licks per bout at the sweet bottle compared to the water bottle (F(1,7) = 57.92, p = 0.0001), which was not found to be significantly affect by experimental manipulations (F(1.817,10.45) = 2.57, p = 0.13). G, Across all experimental manipulations, mice performed more quick switches (<60 s) from water to sucrose than sucrose to water (G, F(1,7) = 14.20, p = 0.0070). H, Experimental manipulations affected memory performance (H, F(2.416,15.70) = 7.235, p = 0.0043) where replacing sucrose with sucralose resulted in reduced returns to water fo [file enu-eN-NWR-0195-23-s06.tif]

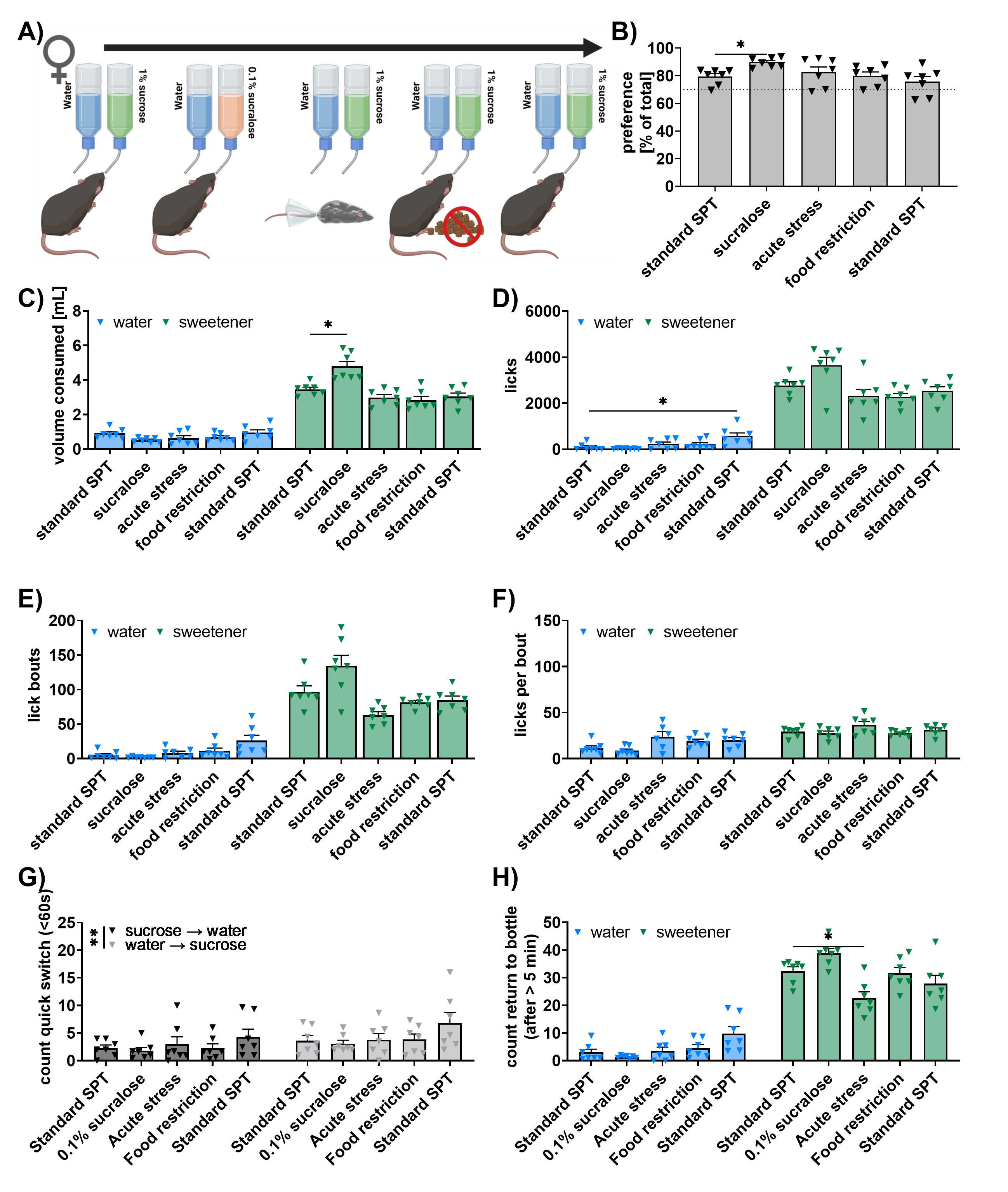

Supplement: Extended Data Figure 6-2 — Noncaloric sweetener increases preferential consumption and acute stress impairs memory performance in female mice. A, Following a standard SPT, female mice underwent three preference tests to measure effects of caloric content and acute stress on drinking behavior. First, 1% sucrose was replaced with 0.1% noncaloric sucralose. Second, sucrose preference was tested on nights following acute multimodal stress. Third, sucrose preference was tested while food was restricted to approximately 50% of normal consumption. Finally, mice were re-tested on the standard SPT. B, Mice exhibited an increased preference for 0.1% sucralose than 1% sucrose over water (B, Friedman test p = 0.0045, Z = 2.70, p = 0.027) but neither acutes stress nor food-restriction affected sucrose preference. C, D, Experimental manipulations also affected the ratio of sucrose versus water drinking as seen in volume consumed (C, F(2.206,13.24) = 19.66, p < 0.0001) and total licks (D, F(1.891,11.34) = 6.277, p = 0.016) with mice consuming more sweetener and when sucrose is replaced with sucralose (C, t = 4.12, p = 0.025) and exhibiting more licks at the water bottle upon retesting under standard conditions (D, t = 3.93, p = 0.030). E, Experimental manipulations also affected the number of lick bouts mice initiated at the water or sweet bottle (E, F(2.574,15.44) = 13.42, p = 0.0002) although a post hoc analysis found no significant differences in lick bouts produced across the experimental manipulations. F, Mice consistently produced more licks per bout at the sweet bottle compared to the water bottle (F(1,6) = 105.6, p < 0.0001) and this was not significantly affected by experimental manipulation (F(1.979,11.38) = 2.414, p = 0.13). G, Across all experimental manipulations, mice performed more quick switches (<60 s) from water to sucrose than sucrose to water (G, F(1,6) = 24.12, p = 0.0027). H) Experimental manipulations affected memory performance (H, F(2.240,13.44) = 7.35, p = 0.0059) where acute stres [file enu-eN-NWR-0195-23-s07.tif]

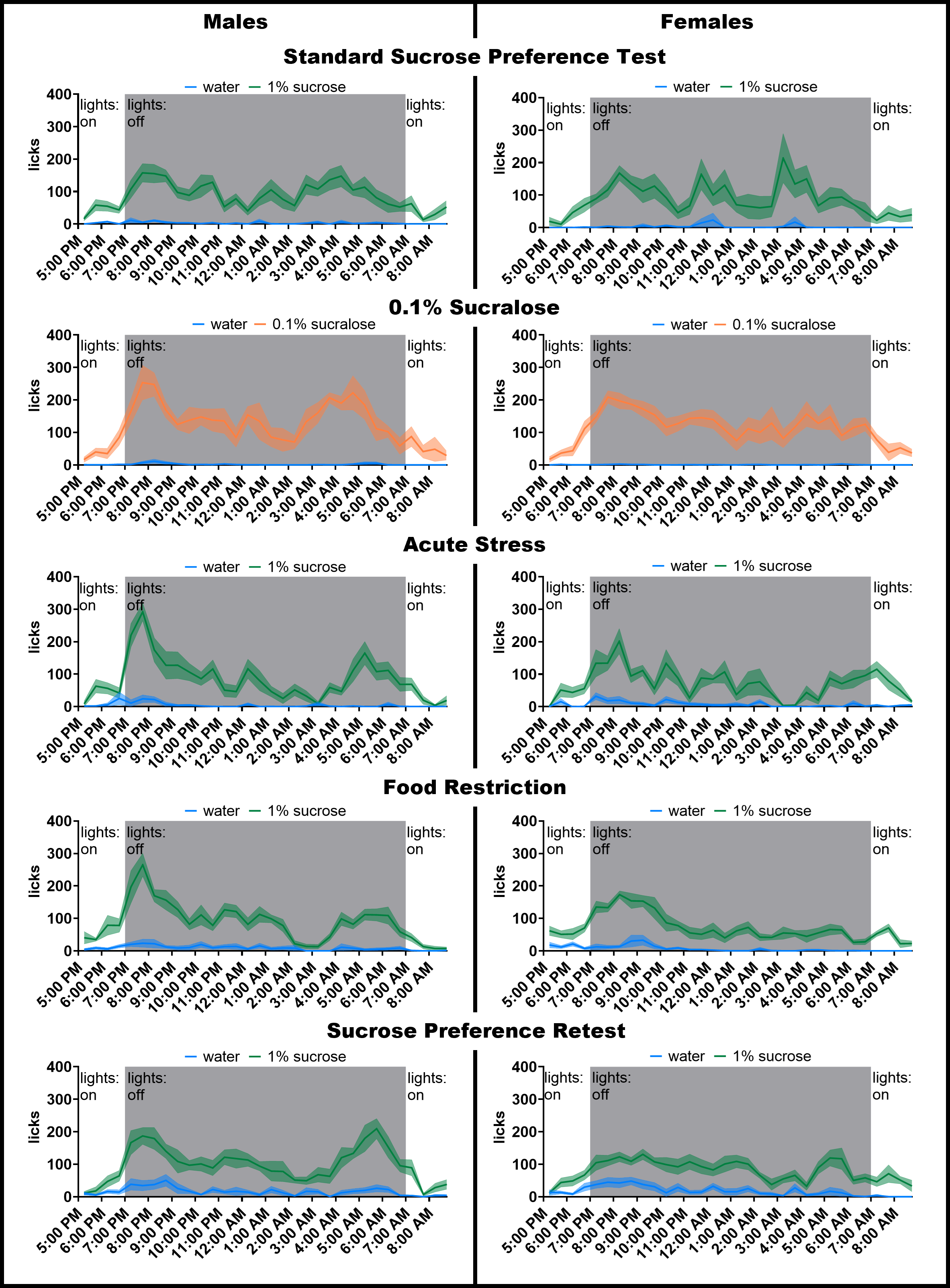

Supplement: Extended Data Figure 6-3 — Circadian pattern of drinking is more robust in males than females and is disrupted by acute stress. Pattern of drinking during standard sucrose preference (A, B), when sucrose is replaced with sucralose (C, D), after mice have been subjected to acute stress (E, F), while mice are undergoing mild food restriction (G, H), and upon retesting under standard conditions (I, J) in male (left column) and female (right column) mice. Download Figure 6-3, TIFF file. [file enu-eN-NWR-0195-23-s08.tif]
